# Biodigesters for Sustainable Food Waste Management

**DOI:** 10.3390/ijerph22030382

**Published:** 2025-03-06

**Authors:** Jay N. Meegoda, Charmi Chande, Ishani Bakshi

**Affiliations:** Civil and Environmental Engineering, New Jersey Institute of Technology, University Heights, Newark, NJ 07102, USAishanibakshi73@gmail.com (I.B.)

**Keywords:** food waste, biodigester, anaerobic digestion (AD), biogas, renewable energy, fertilizer

## Abstract

The global challenge of food waste management poses severe environmental and public health risks. Traditional disposal methods, such as landfilling and incineration, exacerbate these issues. Decomposing food waste in landfills emits methane, a greenhouse gas 25 times more potent than CO_2_, while landfill leachate contaminates soil and groundwater with hazardous pathogens and toxins. Additionally, improper waste disposal fosters microbial proliferation, posing severe health risks. Incineration, though commonly used, is inefficient due to the high moisture content of food waste, leading to incomplete combustion and further air pollution. Therefore, this review examines biodigesters as a sustainable alternative to traditional food waste disposal, assessing their effectiveness in mitigating environmental and health risks while promoting circular economy practices. It evaluates different biodigester designs, their operational scalability, and their economic feasibility across diverse global contexts. Through an analysis of case studies, this review highlights biodigesters’ potential to address localized waste management challenges by converting organic waste into biogas—a renewable energy source—and nutrient-rich digestate, a valuable natural fertilizer. The process reduces greenhouse gas emissions, improves soil health, and minimizes public health risks associated with microbial contamination. Various biodigester designs, including fixed-dome, floating-drum, and tubular systems, are compared for their efficiency and adaptability. Additionally, this review identifies key barriers to biodigester adoption, including feedstock variability, maintenance costs, and policy constraints, while also discussing strategies to enhance their efficiency and accessibility. This review is novel in its comprehensive approach, bridging the technological, environmental, and public health perspectives on biodigesters in food waste management. Unlike prior studies that focused on isolated aspects—such as specific case studies, policy analyses, or laboratory-scale evaluations—this review synthesizes the findings across diverse real-world implementations, offering a holistic understanding of biodigesters’ impact. By addressing knowledge gaps in terms of health risks, environmental benefits, and economic challenges, this study provides valuable insights for policymakers, researchers, and industry stakeholders seeking sustainable waste management solutions.

## 1. Introduction

Addressing the escalating environmental and public health issues associated with food waste disposal is a growing global challenge. In developed nations, food waste has reached unprecedented levels, with an estimated 61% of all food produced going uneaten and contributing to significant environmental degradation [1]. Specifically, approximately 2.5 billion tons of food is wasted every year [2], of which 60 million tons originated in the United States. In other words, the US wastes around 40% of its entire food supply and generates around 325 lbs. of waste per person [3]. The annual food loss in the United States is equivalent to 170 billion kilograms (1.7 × 10^11^ kg) of carbon dioxide (CO_2_), corresponding to approximately 170,000 µmol of CO_2_ per mol of emissions. This amount is comparable to the CO_2_ emissions from 42 coal-fired power plants or 37 million cars [4].

When improperly managed, this waste decomposes in landfills, leading to the emission of greenhouse gases, the contamination of water supplies, and the proliferation of disease vectors, all of which pose severe health risks to nearby communities. Landfill storage of food waste fosters the growth of microbial contaminants such as *Salmonella*, *E. coli*, and *Listeria*, creating health risks that extend beyond the landfill to impact urban and rural communities alike [5].

Food waste significantly contributes to public health risks, encompassing a variety of organic materials, each with specific implications for microbial contamination and environmental harm. Meat and poultry waste often harbor pathogens such as *Salmonella*, *Campylobacter*, *Listeria monocytogenes*, and *Escherichia coli* (*STEC*), which are responsible for illnesses like salmonellosis, listeriosis, and hemolytic uremic syndrome (HUS) [6]. Decomposing meat, especially chicken and beef, provides a conducive environment for these bacteria, posing severe health risks, particularly to immunocompromised individuals and pregnant women [7]. Similarly, dairy products like expired milk and cheese are reservoirs for pathogens such as *Listeria monocytogenes*, *Staphylococcus aureus*, and *Brucella* spp., which can cause conditions ranging from mild foodborne illnesses to life-threatening infections like meningitis [8]. Seafood waste, including spoiled fish and shellfish, also presents risks, harboring pathogens like *Vibrio parahaemolyticus* and *Clostridium botulinum*, which can lead to severe diseases such as botulism and acute gastroenteritis. Additionally, plant-based waste such as fruits, vegetables, and grains often carries *E. coli* (*STEC*), *Salmonella*, and *Aspergillus flavus*, causing gastrointestinal infections and, in some cases, producing carcinogenic aflatoxins [9].

Processed food waste and beverages present further challenges [10,11]. Cooked foods that are inadequately stored promote the growth of pathogens like *Clostridium perfringens* and *Staphylococcus aureus*, causing foodborne illnesses marked by abdominal pain and diarrhea [12,13]. Spoiled fruit juices and alcoholic liquids can foster microbial contamination, yeast overgrowth, and environmental toxicity [14,15]. Egg waste, often contaminated with *Salmonella enteritidis*, is a leading cause of salmonellosis, with symptoms such as fever, abdominal cramps, and diarrhea [16]. To mitigate these risks, effective waste management practices are critical.

The environmental hazards of food waste extend beyond air pollution, posing significant risks to soil and water quality through contamination [17]. As food waste decomposes, it generates leachate, a liquid that, when infiltrating surrounding soils, transports pathogens and toxins into groundwater. This contamination poses direct risks to drinking water supplies, with leachate commonly containing harmful organic compounds, heavy metals, and pathogenic microbes that degrade water quality and impair human health. Additionally, food waste in landfills contributes to air quality degradation due to volatile organic compounds (VOCs) such as ammonia and sulfur compounds, which are released during the decomposition process [18]. Together, these compounds contribute to a decline in air quality, with consequences for respiratory health, especially in vulnerable populations living close to waste disposal sites.

**Figure 1:**
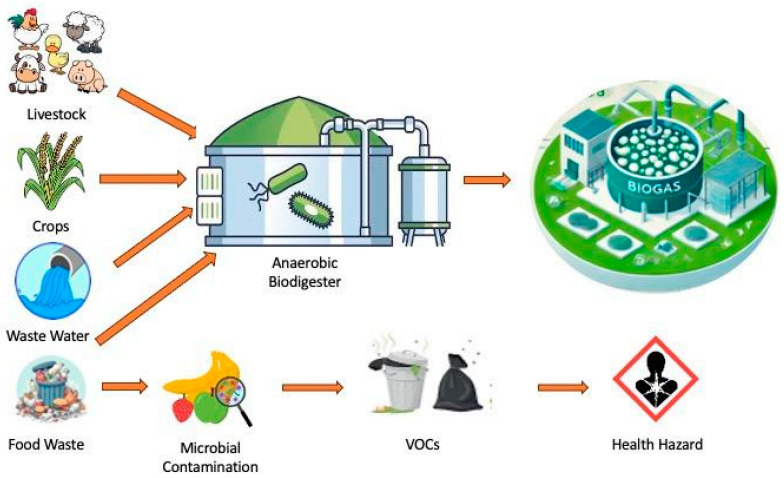
Anaerobic digestion process: inputs, outputs, and toxic effects of food waste.

One increasingly viable solution to mitigate the health and environmental threats posed by food waste is the use of biodigesters. Biodigesters employ anaerobic digestion (AD) to process organic waste in oxygen-free environments, producing biogas—a renewable energy source primarily composed of methane and carbon dioxide—and a nutrient-rich digestate [19]. This digestate can serve as an effective fertilizer, promoting soil health and reducing reliance on synthetic alternatives. Biodigesters offer a sustainable waste management solution, capturing methane emissions that would otherwise escape into the atmosphere, and providing a renewable energy source that can offset fossil fuel use (Figure 1). This dual benefit aligns with global efforts to reduce greenhouse gas emissions and move toward a circular economy. A biodigester acts as a recycling machine with a simple but clever concept [20]. In these containers, microorganisms work constantly to break down waste, produce biogas as energy, and provide valuable nutrients to plants. In addition to solving the problem of waste management, this system promotes environmentally friendly energy production and improves agricultural lands.

Yet, even considering the food waste management potential of biodigesters, there is a lack of comprehensive reviews on their use in a solid waste management setting. Current studies are limited to either a specific case study or performance assessments under ultra-specific conditions, informing readers of the successes and challenges of biodigester-based food waste management in specific regions or conditions such as those surrounding the Valles Oriental Waste Treatment Centre or under the conditions a biogas-fed solid oxide fuel cell system [21,22]. Similarly, the reviews are limited to those on regional policies, such as solid waste management policies in Malaysia or production in Argentina, or on the process and science behind AD, as conducted by the University Polyethnic of Bucharest [23,24,25]. There is a lack of comprehensive reviews of diverse case studies and the health, environmental, and economic effects of real-world implementations of biodigesters for food waste management, which this review intends to address. By analyzing the findings across diverse implementations, this study intends to provide a holistic understanding of biodigester-based food waste management and the resulting environmental and health benefits.

This review aims to elucidate the pressing health and environmental challenges associated with food waste, including microbial contamination, air quality degradation, and the attraction of disease vectors in landfill environments. It then explores the capacity of biodigesters to mitigate these issues, examining how AD can transform waste management practices. Additionally, this paper discusses various biodigester designs and technologies, from small-scale household units to industrial applications, analyzing their operational effectiveness and economic viability across different contexts. This review concludes with a forward-looking perspective on the potential of biodigester technology to revolutionize food waste management, reduce greenhouse gas emissions, and contribute to a more sustainable and resilient food system.

## 2. Health Hazards of Food Waste

### 2.1. Microbial Contamination

Microbial contamination poses significant health risks linked to food waste, an issue of growing concern globally, especially in the United States. When food waste is discarded in landfills, the nutrient-rich environment provides an ideal breeding ground for various microorganisms, including bacteria, molds, and viruses [26]. The landfill environment, with its low oxygen levels and high moisture, creates favorable conditions for microbial growth. Anaerobic bacteria thrive in these settings, breaking down organic material in the waste and releasing methane, along with volatile organic compounds (VOCs), into the air. These gases may carry contaminants and airborne pathogens, posing health risks to nearby communities. Bacteria such as *Salmonella*, *E. coli*, and *Listeria* are known to proliferate in landfill conditions and can lead to various illnesses (Table 1) when humans come into contact with them [27].

### 2.2. Leachate in Landfills

Leachate from contaminated landfill sites is a significant concern when it comes to food waste [34]. Areas around landfills face an increased risk of groundwater contamination due to leachate—a liquid that forms as water interacts with decomposing waste, including spoiled food and other types of municipal solid waste. As anaerobic bacteria break down food waste, leachate can carry harmful microbes that, when reaching groundwater, pose serious health risks to those relying on unfiltered water sources [35]. In addition to harming the soil and surrounding environment, leachate can alter the pH levels of drinking water and contribute to the formation of trihalomethanes (THMs), toxic compounds that negatively impact human health. Reducing food waste in landfills through waste diversion strategies, such as biodigesters, can help limit the production and leakage of leachate, ultimately decreasing the risk of microbial contamination of water supplies [27].

### 2.3. Air Quality Degradation

**Figure 2:**
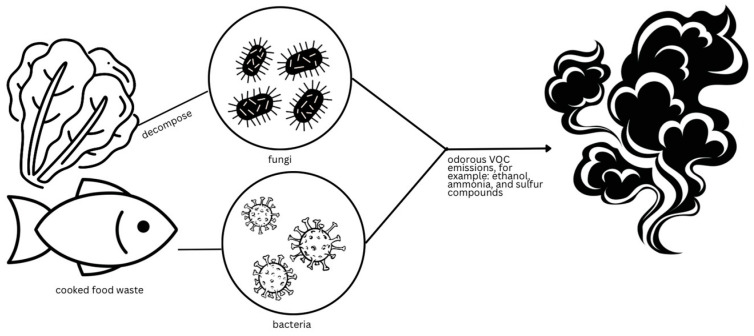
The decomposition of food waste emits volatile organic compounds.

Food waste significantly contributes to global air quality degradation, posing serious health risks (Table 1) [36]. Each year, over one billion tons of food is wasted, much of which decomposes in landfills, releasing various greenhouse gases [37]. Among these, carbon dioxide (CO_2_), methane (CH_4_), and hydrogen sulfide (H_2_S) are the primary emissions. Methane, though present in smaller amounts than CO_2_, has a warming potential that is 281 times stronger, making it a particularly impactful greenhouse gas. In addition to CO_2_ and methane (Figure 2), the decomposition of food waste emits volatile organic compounds (VOCs) such as ethanol, ammonia, and sulfur compounds, all of which contribute to air pollution and further degrade air quality [38].

### 2.4. Attraction of Disease Vectors

Various disease vectors can be attracted to the billions of tons of food that end up in landfill annually. When food decomposes in landfills, it releases odors that are highly attractive to animals, such as rodents, flies, and mosquitoes (Table 2). Many of them carry and transmit diseases to humans through pathogens [39,40].

## 3. Environmental Impact of Food Waste Concerning Municipal Solid Waste (MSW)

In the United States, municipal solid waste (MSW) has increased from 237.77 million metric tons in 2015 to 242.94 million metric tons in 2017, roughly 22% of which is food waste. Food waste within MSW poses myriad environmental complications, including but not limited to increased methane emissions, soil degradation, and water contamination [19]. Food waste is a major component of the organic fraction of municipal solid waste, which leads to MSW methane emissions [41]. Among food waste, proteins and lipids have the highest methane potential, and approximately 50% of all landfill gas is composed of methane [42]. Moreover, a large portion of methane generated by food waste in the landfill escapes landfill gas collection systems and is, in turn, released to the environment. Food waste generates methane through decomposition under anaerobic conditions. According to the US Environmental Protection Agency (EPA), food waste releases 34 tons of methane emissions for every 1000 tons of it landfilled. With methane being a short-lived greenhouse gas compared to CO_2_, reductions in methane generation would have a quick and significant effect [43].

It is key to note that, as per the EPA’s Waste Reduction Model, if 75% of US landfilled food waste was redirected to an anaerobic digester or a composting facility, the overall emissions would show an 80–90% decrease [44]. Additionally, projections concerning global anthropogenic methane emissions claim that in 2020, the landfilling of solid waste generated 40.72 tetragram (Tg)CH_4_, while all biomass combustion processes, a fraction of which comprises biogas generation from food waste management, generated 10.24 TgCH_4_. In 2030, those numbers are projected to be 43.34 and 10.91 TgCH_4_, respectively. For context, the total methane emissions were estimated at 373.56 in 2020 and 405.81 in 2030 [45].

**Figure 3:**
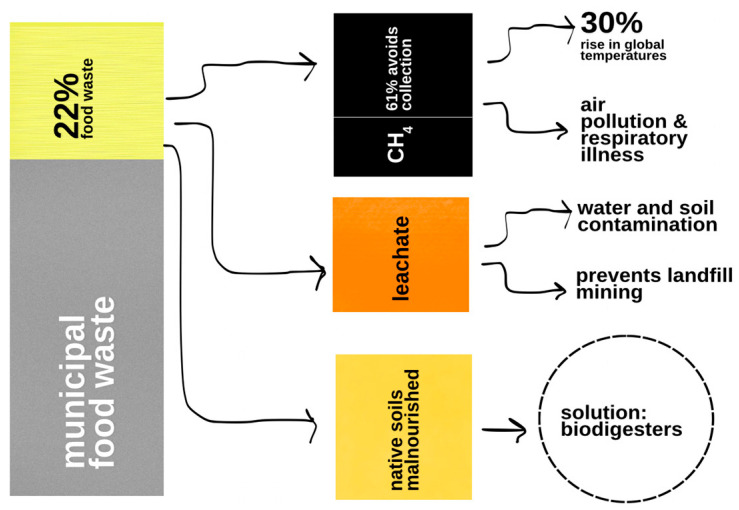
Municipal solid waste (MSW) leads to environmental side effects.

Additionally, food waste majorly contributes to leachate, a liquid that drains from landfill and contaminates groundwater. Typically, landfills have specialized drainage systems in place to manage their leachate, but the concentration/toxicity of contaminants, the type of geologic strata, and the direction of groundwater flow can lead to leachate seeping into the soil. Once leachate contaminates groundwater, a plume of contamination is created. Pathogens, such as *leptospirosis*, found in food waste can spread into soil and water [34]. Excess nutrients from these wastes lead to harmful algal blooms and nitrate contamination of drinking water, which has been known to cause hyperthyroidism and blue baby syndrome [43]. In general, over 27 metric tons of food waste finds itself in the depths of landfills, which equates to around 21 trillion liters in the liquid phase (Figure 3) [19]. Without the contamination brought about by food waste, landfill mining—the excavation of recyclables such as paper, glass, metal, etc.—could be possible. In addition, redirecting food waste to landfills disadvantages urban soils. Typically, urban soils are not uniform in their properties, combine with non-native soils, boasting low nutrient levels, low organic matter, and high soil compaction. The need for healthy soil is great, as healthy soil absorbs carbon from the atmosphere. Even now, the European Commission reports that 73–79 billion tons of carbon in the continent’s soil. With the assistance of compost, the soil’s textures will transform to better retain nutrients and moisture, sequester carbon, and prevent erosion [46].

Specifically, the implementation of biodigesters would mitigate the greenhouse gas (GHG) emissions of MSW, improve soil fertility, conserve resources, and prevent the other detrimental effects brought about by food waste. By capturing methane during the AD process, biodigesters prevent the release of this potent greenhouse gas into the atmosphere, mitigating climate change. It is estimated that the methane emissions reduction involving biogas digestors can avoid 0.25 °C of global warming by the mid-century. It is key to consider that agriculture extraction and processing, and coal mining, have almost the same amount of methane emissions [47]. Overall, biogas can decrease global emissions through its electricity generation by 18–20% [48], and in general, over a life cycle, biogas can reduce greenhouse gas emissions 90% more effectively than fossil fuels [49]. Specifically, it is estimated that if all 105 billion tons of global organic waste was recycled as biogas, the GHG emissions would be reduced by 10% and 50% of the Global Methane Pledge could be delivered by 2030 [50].

The nutrient profile of biodigester digestate is influenced by the composition of the feedstock and the operational parameters of the anaerobic digestion process. The total nitrogen (N) concentration in the digestate typically ranges from 1.5% to 6.0%, with ammonium nitrogen (NH_4_-N) accounting for approximately 0.3% to 2.0%, providing a readily available nitrogen source for plant uptake. The phosphorus (P) content generally falls within 0.2% to 2.5%, supporting root development and energy transfer in plants. Similarly, the total potassium (K) concentration varies from 0.5% to 5.0%, contributing to plant stress resistance and overall soil fertility. In addition to macronutrients, the digestate contains organic matter in the range of 30% to 60%, which enhances the soil structure and microbial activity. Furthermore, the moisture content remains high, typically 70% to 95%, allowing for easy application as a liquid biofertilizer and facilitating nutrient assimilation in soil ecosystems [51]. It can also convert organic matter into an odorless bio-slurry, which serves as a fertilizer and can prevent nitrate water contamination. The digestate can increase soil fertility, which increases crop yields, facilitate permaculture practices and support sustainable agriculture practices as agriculture requires healthy soil, water management, minimized air/water pollution and carbon source. Digestate enables the recycling of nutrients within ecosystems and improves the resilience of agricultural systems. Overall, though posing myriad environmental concerns, food waste can be largely mitigated by implementing biodigesters.

## 4. Biodigesters as a Solution to Health and Environmental Hazards

Biodigesters offer a powerful solution to the numerous health and environmental hazards posed by food waste, particularly in large urban areas where waste accumulation is pronounced. Through the process of AD, biodigesters effectively decompose organic waste in an oxygen-free environment, generating biogas and digestate, both of which have valuable applications [52]. Biogas, a renewable energy source primarily composed of methane, can be captured and used for electricity generation, heating, or even as a fuel for vehicles. This conversion of waste into usable energy helps reduce the demand for fossil fuels, contributing to lower greenhouse gas emissions and promoting a more sustainable energy landscape.

### 4.1. Microbial Interaction, Process Optimization, and Biogas Yield in Anaerobic Digestion

Anaerobic digestion (AD) is a multi-step process driven by various microorganisms, which convert organic matter into biogas and digestate [20]. The process occurs in four sequential stages: hydrolysis, acidogenesis, acetogenesis, and methanogenesis. In a typical single-stage batch reactor, all four processes take place in the same vessel [19]. **Hydrolysis:** Complex organic materials such as carbohydrates, proteins, and fats are broken down into simpler molecules like sugars, amino acids, and fatty acids by hydrolytic bacteria. This step is critical as it transforms large polymers into forms that can be utilized by microorganisms in the subsequent steps. **Acidogenesis:** Acidogenic bacteria metabolize the products of hydrolysis, producing volatile fatty acids (VFAs), alcohols, and gases like hydrogen and carbon dioxide. While this stage occurs rapidly, excessive production of VFAs can lower the pH, potentially inhibiting the overall digestion process. **Acetogenesis:** Acetogenic bacteria convert VFAs and other intermediate products into simpler compounds, primarily acetate, hydrogen, and carbon dioxide. This step is essential for preparing the substrates for methanogenesis. Hydrogenotrophic methanogens help maintain favorable conditions by consuming hydrogen, preventing the inhibition of acetogenic bacteria. **Methanogenesis:** In the final stage, methanogenic archaea convert acetate and hydrogen into methane and carbon dioxide, the primary components of biogas. Methanogenesis is highly sensitive to environmental conditions, including the pH and temperature, and is often the rate-limiting step due to the slow growth rate of methanogens. Understanding the interactions between these microbial communities is critical to optimizing biogas production. Improvements in reactor design and process conditions, such as temperature control and pH regulation, can significantly enhance the efficiency of the AD process, making it a valuable tool for sustainable waste management [53].

### 4.2. Operational Procedures and Mechanisms

Biodigesters function through the four-step process of AD, which requires a certain composition of organic and seed material input, along with preferable conditions for the bacteria to process and break down the matter. As the digestion process occurs, the by-products of methane-rich biogas and nutrient-rich digestate are produced.

#### 4.2.1. Organic Loading Rate

The AD process requires the starting organic material to feed the bacteria so the matter can be broken down and the by-products can be produced. The organic material is fed through an inlet in the biodigester and can include a mixture of food waste, farm waste, animal manure, wastewater sludge, and other biodegradable materials. Certain compositions and ratios of bacteria-rich seed cultures and food waste compositions will allow for a more diverse and efficient digestion process, which will be discussed in a later section. Commonly, the organic waste must be pretreated before insertion into the digester. It is important for the organic matter to be a homogenous mixture and ground as small as possible to provide an ideal feed for the hydrolytic bacteria to break down the polymers of the food waste into their simple monomer structures. Various methods, such as putting the organic waste through a grinder, including a mixer in the digester, or crushing food particles into smaller components using ultrasound technology, have shown potential to expedite the rate-determining step of hydrolysis [54]. It is also important to ensure that the organic material inserted into the digester is not contaminated by non-degradable materials such as rock, glass, plastic, or other contaminants that hinder the digestion process. Once pretreated, the organic material must be diluted with a liquid source such as water, wastewater sludge, or recirculated liquid from digestion effluent material [54].

The loading rate of the biodigester depends on the capacity of the organic materials the digester can be fed per day and is determined by the efficiency of the bacteria contained in each successive step of the digestion process. If too much organic material is loaded into the digester at once, it can lead to the overproduction of VFAs, which hinders the ability of methanogenesis, reducing the amount of methane produced [20]. Diversifying the microorganisms or supplying the digester with rapid shocks of loading material can train the digester to intake organic feed at a higher rate, allowing the feeding capacity to increase and, hence, expediting the digestion process as a whole. The optimal operational conditions for anaerobic digestion (AD) of food waste are influenced by the physicochemical properties of the feedstock and the specific reactor design. The organic loading rate (OLR) typically falls within the range of 1.0 to 5.0 kg volatile solids (VSs) per cubic meter per day, as exceeding this threshold can result in an excessive accumulation of VFAs, leading to process acidification and inhibition of methanogenic activity. Maintaining a moisture content between 70% and 95% is critical to sustaining microbial consortia and facilitating the hydrolytic enzyme activity necessary for substrate degradation. Additionally, an optimal mixing rate of 10 to 50 revolutions per minute (rpm) enhances microbial–substrate interactions, prevents phase separation, and ensures uniform temperature and pH distribution within the digester. However, excessive agitation may disrupt the syntrophic relationships between hydrolytic, acidogenic, acetogenic, and methanogenic consortia, while insufficient mixing may lead to stratification and localized substrate inhibition, thereby reducing the overall biogas yield and system stability [55].

#### 4.2.2. Anaerobic Digestor Tank

Once the organic waste enters the biodigester, it is stored in an AD tank that creates an ideal environment for the digestion process to occur. To create an ideal environment for the digestion process, a few important factors must be considered. The bacteria work most efficiently in a dark environment, so it is important to eliminate light exposure in the tank. The tank must also be void of oxygen since the process is anaerobic. The temperature of the system must also be controlled to optimize digestion. There are two optimal temperatures for AD: the optimal mesophilic range is between 30 and 35 °C, while the optimal thermophilic range is between 50 and 65 °C [54]. Mixing in the AD tank may also help increase the digestion speed by combining the fresh organic waste with the digestate-containing microorganism, preventing scum formation and supporting a homogenous temperature throughout the system. Too much mixing can lead to digestion disruption, so it is best to mix the contents at a slow speed. The digestion tank must also provide the proper pH level for the AD process. The different stages of AD prefer different pH levels, and it is important to monitor and adjust the pH conditions of the tank as needed throughout the process. The ideal pH level for the entire process is between 5.5 and 8.5 [54]. Biodigesters can either be single-stage or multi-stage. Single-stage digesters consist of one reactor and all the stages of digestion occur sequentially in one tank, while multi-stage digesters consist of multiple tanks and perform the various steps of digestion in separate tanks, also known as one-stage anaerobic digestion and two-stage anaerobic digestion, respectively [54,56,57]. Separating the stages of digestion may have benefits since the stages prefer different pH levels to optimize their bacterial processes, so the separation allows for individualized pH monitoring.

#### 4.2.3. Microbial Decomposition and Optimization

Microbial decomposition occurs sequentially in the first three steps of the process: hydrolysis, acidogenesis, and acetogenesis. The overall decomposition process occurs through the cooperation of a diverse spectrum of microorganisms that thrive in an anaerobic environment and exhibit the characteristics necessary to convert the substrates from organic matter into biogas and digestate. Previous studies have found that optimized microbial communities are dominated by the bacteria *Ruminococcus* sp. and *Clostridium* sp. and the archaea *Methanoculleus* sp. [58]. More specifically, optimized microbial cultures require manipulation of the environmental conditions, which in turn enriches the functional microbial populations involved in the three stages of AD. For instance, extending the hydraulic retention time and reducing the mixing intensity can promote the growth of hydrolytic bacteria such as *Clostridium* and *Bacteroides*. Gradually increasing the organic loading rates favors acidogenic fermenters, enhancing the substrate availability for methanogens. Thermophilic conditions have also been shown to improve biogas yields by favoring specific methanogenic archaea like *Methanothermobacter*, though the energy costs must also be balanced [33]. Additionally, optimizing syntrophic interactions by controlling the substrate availability and dilution rates in sequencing batch reactors can strengthen the interdependencies between acetogenic bacteria and methanogens, preventing process imbalances. Further monitoring of microbial community shifts through high-throughput sequencing can help refine the operational parameters and cultivate highly efficient, substrate-specific microbial communities for enhanced bio-digestion [59].

#### 4.2.4. Inoculation Optimization

Inoculation—the adding of microorganisms (seed sludge) to a digester to accelerate the breakdown of organic matter—is key to maintaining stability in dry AD, including that of food waste. Common inoculum sources such as activated sludge, manure, and digestate from wet or dry AD with enriched methanogenic populations accelerate biochemical reactions, reducing the digestion time [60]. Studies have proven the inoculum-to-substrate (I/S) ratios to significantly affect AD performance, with one study identifying that a 1:4 I/S ratio generated a lower methane yield and longer lag-phase time than a 1:1 I/S ratio [61]. However, there is no one ideal I/S ratio, as the system conditions and feedstock characteristics have a major influence. In combination with inoculation, pretreatment strategies, such as mechanical, chemical, biological, and thermal treatments, have also been proven to enhance AD performance [60,62].

#### 4.2.5. Biogas Production

During the final stage of AD, methanogenesis, methanogenic microorganisms consume the intermediates produced in the previous steps to create biogas. The biogas produced is a mixture of methane, carbon dioxide, and other inert gasses [54]. The biogas production time varies from system to system and can range from 10 to 40 days. Factors such as the temperature, added technologies, and waste composition can alter the retention time [54]. The biogas created can be captured and stored to be utilized. The tank can be connected to a gas pipe and pressurized to create a usable energy source to power heating, cooking, and electricity [20]. The other by-product, apart from biogas, is a rich slurry called digestate. The digestate can be stored and used in farms, while the excess effluent waste can be discharged into sewers or reused as the liquid component for the initial input of bio-digestion.

The design of biodigesters allows them to operate continuously. New organic waste material must be constantly inserted into the system, and as it is broken down, the by-products must be removed and utilized. This creates a regenerative and sustainable waste management system. Maintaining the biodigester properly ensures the maximum efficiency and stability of biogas and digestate production.

## 5. Biogas and Digestate Utilization

**Cooking:** Biogas can be used for cooking, replacing traditional cooking fuels such as wood, coal, or liquefied petroleum gas (LPG). Overall, biodigesters can be installed in homes to produce biogas for cooking, heating, and lighting. This provides clean, renewable energy for daily household operations, reduces dependence on fossil fuels, and improves indoor air quality.

**Heating:** Biogas can be used for space heating, water heating, and even in industrial processes requiring heat. Biogas plants can utilize a portion of their produced biogas for future process heating, further increasing the self-sufficiency and sustainability of biogas production [63]. Boilers are reliable applications with low requirements in terms of biogas quality and minimal treatment through desulfurization and pressure monitoring [64].

**Electricity generation:** Biogas can power generators to produce electricity, offering a renewable energy source for homes and businesses. Biogas can act as fuel for power/combined heat and power (CHP) generation technologies such as internal combustion (dual-fuel and spark-ignition engines), gas turbines, and hot fuel cells [64,65]. Community-owned or managed bio-digestive systems support local energy production.

**Vehicle fuel:** Biogas can be refined and used as a vehicle fuel, reducing reliance on fossil fuels. Natural gas vehicles (NVGs) can emit 95–99% less carbon dioxide compared to vehicles with diesel or petrol engines [64].

Digestate is composed of indigestible material and dead microorganisms. Using digestate as an alternative to synthesized fertilizer, which is extracted from natural gas, can decrease the use of fossil fuels, lower the greenhouse gas emissions, and save energy for other uses. “Digestate or slurry from the digester is rich in ammonium and other nutrients used as an organic fertilizer which include phosphorus and potassium” [17]. With the mineral-rich post-digestion material, it can be returned to be used in the environment as a substitute for synthetic fertilizers. Among its benefits as a fertilizer, slurry mixed in tandem with plant material is significant for earthworms [17]. Digestate can be used as is and spread on land to serve as a fertilizer.

Current studies showed that as opposed to conventional fertilizers, digestate fertilizers form microbial populations with diverse ecological profiles. More specifically, at the phylum level, the microbial communities remained unchanged between conventional and digestate treatments, but the digestate did introduce new genera involved in nutrient cycling and carbon storage. However, the resultant stochastic microbial structures led to variable microbial community responses. In contrast, conventional fertilizers form structured microbial communities, promoting specific nitrogen-associated taxa, though they do not contribute to microbial diversity as much and can, over time, disrupt soil ecology. Therefore, the studies concluded that a mixed fertilizer could temper the extremity of digestate fertilizer while still reaping its benefits [66].

Alternatively, it can be separated into liquor and fibers, which have different compositions of minerals and nutrients, as described by the Official Information Portal on AD in the United Kingdom. The liquid can be used for crop growth and the fiber can also be applied to the soil or stabilized for making compost. Digestate, as explained by the US EPA, comprises the materials left over from the digestion process and can be turned into bedding for livestock, along with flowerpots, on top of fertilizer-related uses. Digestate, in addition to be applied on the soil, can be packaged and sent to stores for other consumers to buy for their own needs. While these are current approaches, the EPA-mentioned technologies are becoming more advanced and can potentially be used on post-digested material to extract the nitrogen and phosphor in digestate to form “concentration nutrient product” like struvite.

## 6. Microbial Sources for Biodigesters

The microorganisms necessary for AD mainly come from various organic wastes and often thrive in specific conditions. Specific strategies for optimizing microbial consorts were elaborated upon in Section 4.2.3 and the interested reader can reference [58,59] for more detailed discussions on optimizing microbial consortia for higher biogas yields. Continuing on, the co-digestion of multiple organic wastes can increase the methane yield due to increased microbial diversity, which can create positive synergisms within the digestion medium and allow for co-substrates to supply nutrients that would not be present otherwise [5]. To enhance the microbial diversity within a biodigester or similar anaerobic system, several strategies can be employed to introduce or enrich functionally diverse microbial communities. The potential sources of microbial inoculum include wastewater treatment plants and sewage sludge, which contain established anaerobic microbial consortia capable of efficient organic matter degradation [67]. Composting facilities serve as another viable source, as they harbor a broad range of hydrolytic and fermentative microorganisms involved in organic waste decomposition. Additionally, livestock manure is a rich reservoir of methanogenic archaea and fermentative bacteria that can accelerate the adaptation of microbial populations within the digester [10].

Natural ecosystems such as freshwater bodies, sediments, and wetlands also support diverse microbial communities that contribute to anaerobic digestion processes. Landfills, particularly anaerobic sections, provide microbial consortia adapted to decomposing complex organic waste under low-oxygen conditions. For more controlled or targeted microbial enhancements, cultures can be sourced from scientific suppliers, university microbiology departments, or specialized culture collections that offer well-characterized strains for biogas optimization. Consulting microbiologists or environmental biotechnologists can further aid in selecting, enriching, and maintaining microbial consortia best suited for the specific bio-digestion process [11].

Specifically, compost leachate, a liquid produced by water draining over compost, has a high organic content and is readily available as a biogas seed material. Though typically acidic, it can be balanced. Studies have found that leachate can have a biogas production up to 9.3 L per liter of reactor volume per day with 70–78% methane content, promoting a satisfactory yield. However, compared to the other feedstocks, compost produces less biogas per unit volume [68]. On the other hand, wastewater septic tank sludge produced from wastewater treatment plants can undergo methanation to produce biogas. Specifically, wastewater contains high levels of organic matter, which makes it fit for AD. By converting wastewater sludge into biogas, the biological and chemical oxygen demands of the wastewater are reduced, as AD breaks down organic matter that would otherwise require decomposition via aerobic microorganisms or be oxidized (biogas from wastewater treatment plants). However, compared to manure, septic tank sludge has lower stability (measured by significant drops in the pH and higher VFA concentrations) and lower biogas yields [69]. The biogas production efficiency varies across different feedstocks due to differences in the organic composition and biodegradability. Food waste exhibits one of the highest biogas yields, ranging from 100 to 200 m^3^ per ton, with a methane content of 60–70%, making it a highly effective substrate for anaerobic digestion. In comparison, animal manure produces 20–40 m^3^/ton with a methane content of 55–65%, while sewage sludge generates a lower yield of 10–20 m^3^/ton, albeit with a similar methane concentration of 60–70%. Agricultural residues contribute 50–100 m^3^/ton, whereas energy crops can yield up to 150–200 m^3^/ton, offering a competitive alternative for bioenergy production. Integrating co-digestion strategies, particularly by combining food waste with manure or agricultural residues, can enhance the process stability and optimize the methane output. Given its high organic load and degradability, food waste remains a promising feedstock for biogas production, providing a sustainable approach to renewable energy generation while mitigating organic waste accumulation [12,70]. Last, cow, poultry, beef and hog manures, due to the high organic content and methane potential, serve as effective biogas feedstock material. If left unutilized, the nitrogen and phosphorus contents in manure can contact with water bodies, posing environmental and health risks. Therefore, many opt to use manure as feedstock for biogas, as it has a neutral pH (high stability), a high buffering capacity, and a naturally occurring composition of nutrients microbes fit for AD [71]. Previously conducted studies have found that manure has the highest cumulative biogas yield of all the feedstocks and maintains the most stability, as witnessed by the optimal pH and alkalinity levels [69]. Other studies have found that of all the strains of manure, cow dung is the most effective, as it boasts the highest methane content and biogas output [72]. However, it is key to note that the potential presence of pathogenic bacteria in manure might pose challenges.

To assess the seed material potential, biochemical methane potential (BMP) assays can estimate the biogas yield of feedstocks. For a minimum of thirty days, organic material is mixed with inoculum and placed in sealed, anaerobic conditions, and incubated to allow the production of biogas. The biogas and its methane content are regularly measured, with the methane yield typically expressed as the volume of methane per unit of volatile solids or additional chemical oxygen demand. Additionally, gas chromatographs can be added to the assays to determine the specific concentrations of methane, carbon dioxide, etc. Overall, these assays can be used to compare different seed materials (biogas from wastewater treatment plants).

When introducing bacteria from these natural sources, maintaining a balanced microbial ecosystem is crucial to the biodigester’s efficiency, emphasizing the importance of gradual and careful introductions. Failure to do this can be costly, both due to disrupting the energy supply and due to requiring up to several months to re-establish the diverse community of microbes [73]. The stability of microbiomes for AD is therefore desirable and necessary for an optimized biogas yield.

## 7. Types of Biodigesters

### 7.1. One-Stage Anaerobic Digestion

Biodigesters can be categorized under many branches. One of the biggest classifications is whether it is a wet (low solids) or dry (high solids) digester. This refers to the moisture content of the feedstocks, as outlined by the EPA. A wet digester normally has less than 15% solids and is usually in a slurry form, whereas a dry digester has more than 15% solids and its feedstock is “described as stackable” [74]. Another possible distinction is batch and continuous flow digesters. Batch digesters have the feedstock loaded altogether and then the time is set up for digestion to occur. After this time, the digester is emptied and loaded with a new batch of feedstock. A continuous flow digester has feedstock constantly added and digested material removed continuously [74]. Other types of digesters include standalone digesters, on-farm digesters, and wastewater digesters, which serve specific functions based on the needs of the customer.

**Figure 4:**
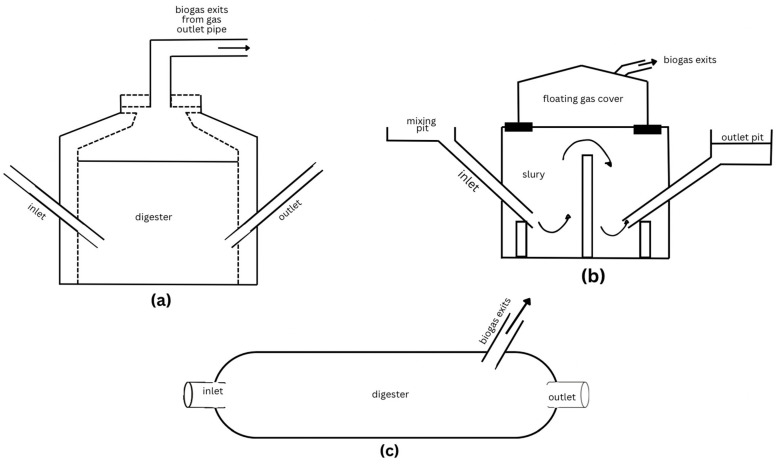
Three major types of digesters in the developing world: (**a**) fixed-dome digester, (**b**) floating-cover digester, and (**c**) balloon or tube digester.

The majority of biodigesters fall into three main types, which are heavily used in developing nations. In Figure 4 below, these include the fixed-dome, floating-cover, and balloon/tube digester. Fixed domes, also called hydraulic digesters, are common in Nepal, Vietnam, Pakistan, and across Africa. Floating-cover digesters are common in India and Africa. Balloon or tube digesters are primarily found in Southeast Asian countries like Vietnam. All three of these types are used in China, but fixed domes represent the majority. Small-scale digesters are mainly used for converting waste to energy in rural areas to provide electricity or heating for families. These digesters are easy to fabricate and use available materials. Such biodigesters have the potential to not only meet the needs of urban residents but also to assist with sanitation management [75].

The fixed-dome biodigester is a semi-batch reactor that is usually created underground as either a pit or manhole and surrounded with reinforced concrete to protect the digester from pressure and to save space, especially in crowded urban areas [76]. It has a fermentation chamber where the organic waste is kept for AD. There is a channel to include feed and a fixed-dome gas holder with iron rods for gas storage, a tank where displaced slurry enters, and an outlet for the biogas to collect into. This digester is originally from China and can be adapted to various climates and waste amounts.

The floating-drum biodigester, which was first designed in India, is cylindrical with a dome and is also stored underground. The difference between the fixed done and the floating drum is that the gas holder in the floating drum is a moving gas holder that hangs over the fermented slurry [76]. The drum is placed upside down and moves based on the volume of the gas inside. There is a frame around the drum, which stops it from toppling over.

The last of the main three types is the balloon or tubular digester, which has a plastic bag placed on a dug trench for safety and is attached to a drainpipe at one of the ends. These pipes serve as the outlet for the discharge of slurry and removal of gas [76]. The bag begins to expand as biogas is generated and is piped away. This model is used throughout African, Southeast Asia, and some Latin American countries.

Another predominant model is a fiberglass digester, which has a digesting and storage part, though there is no barrier between them. This model allows it to be movable and lightweight, and its membrane is “suitable when managing inhibition triggered by ammonia accumulation” [76]. These membrane bioreactors are relatively new in the process of collecting biogas production and operate at 7 on the pH scale. Table 3 shows the data collected and used to compare the main three designs for biodigesters [76,77,78,79].

Classifying biodigesters can be performed based on the method used and the volume of feedstock. Under these conditions, the types are batch, semi-batch, and continuous. Batch reactors are fed slurry and have a specific retention time for digestion and are removed after said time. For a continuous stirred tank reactor (CSTR), it is continuously fed feedstock and products are continuously taken out [76]. The system operates under a wet process mesophilic (moderate temperature with growth around 20 to 45 °C but most ideally under 37 °C).

For semi-batch digesters, look at the anaerobic sequencing batch reactor (ASBR) to understand its properties, alongside the up-flow anaerobic solid removal reactor (UASR), which is a type of plug-flow digester used primarily for the farming industry. The study summarizes its findings in that the better performance experienced with the ASBR when using pumpkin wastewater was due to it being a semi-batch digester under dynamic operating conditions compared to the continuous operating conditions of the UASR reactor [80]. In the pumpkin processing industry, AD is used before aerobic polishing and is better than only using aerobic treatment. Upon using real pumpkin wastewater and synthetic pumpkin wastewater (which was designed to have a constant substrate over a long duration and reduce heterogeneity), it was determined that there are promising methane production rates in the ASBR when using real wastewater samples. Del Agua et al. noted that there was never a concern with reactor stability, which signifies that “high-rate digestion is a feasible approach for solid rich, high strength organic wastes resulting in smaller volume for the high-rate digesters compared with CSTR [80]”. The procedure used for the ASBR involved 6 h between each feeding, which produced methane at a rate of 32.9 mL min^−1^, compared to a continuous methane rate of 12.0 mL min^−1^ for the UASR [80]. Another measurement depicting the advantage of the ASBR over the UASR is the higher solids removal efficiency of the ASBR, which is shown through the higher ammonia concentration in the digestion of real pumpkin wastewater. The study concluded by demonstrating that each method needs to be analyzed to understand its ability to digest the organic waste and utilize the timing and conditions to determine the most appropriate biodigester based on the needs of the customer or industry.

**Figure 5:**
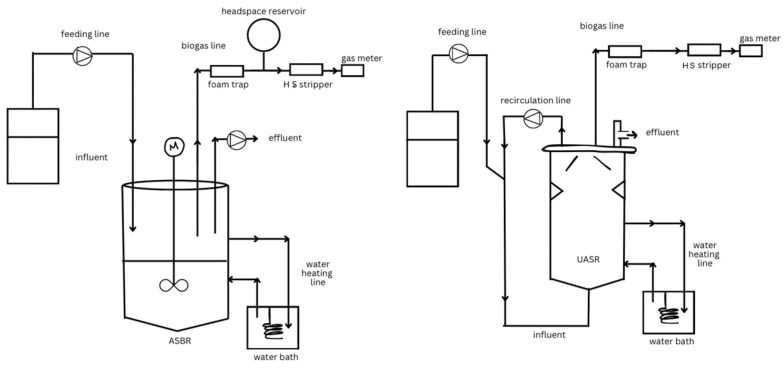
Anaerobic sequencing batch reactor (ASBR) and up-flow anaerobic solid removal reactor (UASR).

While this study highlights how a high-rate digester utilizing a semi-batch approach provides better digestion for this scenario, it should be noted that this is because high-solid wastewater is suited for a semi-batch digester. Del Agua et al. highlighted that UASB reactors are typically used in industrial wastewaters with low concentrations of solids, usually <1% of the total solids and organic compounds [80]. If this study was performed with low-solid wastewater, the UASB would have better performance than in this study and possibly excel over what the ASBR is capable of. Figure 5 illustrates the designs of both biodigesters.

The system included the reactors, feeding reservoirs, feeding pumps, effluent pumps, recirculation pumps, water bath, mixing motor (M), foam traps, headspace reservoir, H_2_S strippers, and gas meters. However, there is no explicit mention that these digesters are designed or optimized for fermenting food waste. Given that food waste digestion requires specific pretreatments, particle size reductions, and optimized organic loading rates, the applicability of these systems to food waste fermentation remains unclear based on the current study design.

**Figure 6:**
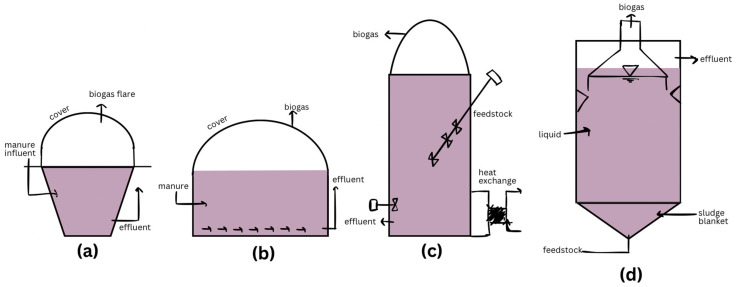
Designs of UASB digesters: (**a**) typical covered lagoon, (**b**) plug-flow digester, (**c**) typical complex mix digester, and (**d**) typical up-flow anaerobic sludge blanket (USAB) digester [81].

Under these main three categories, there are many derivations of digesters, which include the anaerobic contact reactor (ACR), ASBR, up-flow anaerobic solid-state reactor (UASS), up-flow anaerobic sludge bed reactor (UASB), membrane anaerobic system (MAS), modified anaerobic baffled reactor (MABR), and expanded granular sludge beds (EGSB), to name a few [76]. These digesters can be seen across the world with many functionalities, including the following: Morocco uses the UASB to react with recycled papermill wastewater and Nigeria uses the EGSB to digest palm oil mill effluent. Abubakar et al. [76] described how plug-flow systems allow the treatment of high amounts of waste per unit digester volume, require little to no water, and reduce the need for pretreatment. For reference in terms of the above pieces discussing plug-flow and UASB digesters, Figure 6 shows four designs.

Another style of biodigester is a hybrid digester. A hybrid digester is a digester that represents a mixture of other digesters in order to improve the efficiency and performance of the digestion process. A study conducted by Sanchez et al. discussed the building of a vertically constructed wetland and the testing of a hybrid digester (HD) [82]. The HD was created by adding an anaerobic filter (AF) over an up-flow anaerobic sludge bed. Such a design has benefits, which include the high overall biomass concentration, higher resistance to shock loads and toxicity, and reduced capital cost [82]. Previous studies have noted an increase in the removal of organic material. The hybrid digestor–vertical flow (HD-VF) system sought to determine if it was capable of reducing the treatment to two steps and performing a more thorough removal of nitrogen. The HD-VF system achieved high removal efficiency (well into the 90% range) for TSS (total suspended solids), COD (chemical oxygen demand), and BOD (biological oxygen demand), removing most of them, providing evidence in one of the first promising studies on the use of HD-VF systems [82].

### 7.2. Two-Stage Anaerobic Digestion

**Figure 7:**
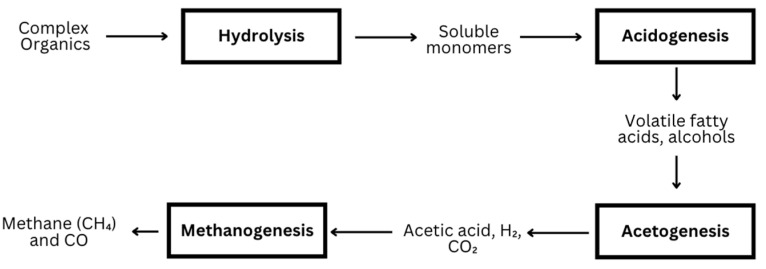
Standard multi-stage AD system.

A two-stage biodigester is a method that can be used to enhance the efficiency and output of biogas production. This system separates the process of AD into two distinct phases: (1) hydrolysis and acidogenesis in the first tank and (2) acetogenesis and methanogenesis in the second tank. The benefit of separating the phases is that each microbial community can operate under optimal conditions. For example, the first tank is heated to improve the efficiency of hydrolysis and acidogenesis, while the second tank is not. Figure 7 details the stages of an anaerobic system.

A two-phase anaerobic digestion system enhances the process efficiency by partitioning the microbial activity into distinct stages, each optimized for specific biochemical transformations. In the first phase, hydrolysis and acidogenesis occur under acidic conditions (pH 5.0–6.5), with temperatures maintained between 35 and 55 °C to accelerate the depolymerization of complex organic substrates into VFAs. This phase has a short retention time of 2–5 days and requires a high moisture content (85–95%) to support the metabolic activity of hydrolytic and acidogenic bacteria. Since methanogenesis is absent from this stage, methane production remains negligible.

The second phase, dedicated to acetogenesis and methanogenesis, operates under neutral to slightly alkaline conditions (pH 6.8–8.2), with mesophilic temperatures (35–40 °C) facilitating the efficient conversion of VFAs into CH_4_ and CO_2_. The retention time extends to 10–20 days, allowing for complete metabolic conversion, while the moisture content is slightly lower (80–90%) to support the growth of acetogenic bacteria and methanogenic archaea. This phase is critical to maximizing the methane yield, as methanogenic archaea are highly sensitive to environmental fluctuations [13].

Compared to single-phase digesters, two-phase anaerobic digestion systems offer distinct advantages, including greater microbial efficiency, as each phase is maintained at conditions that optimize the activity of specific microbial communities [14]. The separation of hydrolysis from methanogenesis prevents the accumulation of acidic intermediates, thereby mitigating the risk of methanogen inhibition and process failure. Additionally, the system facilitates higher methane production rates and greater process stability, as the first phase ensures effective substrate breakdown, allowing for more efficient bioconversion in the methanogenic stage. The ability to regulate the pH, temperature, and organic loading rates independently in each phase further enhances the overall process control, making two-phase digesters more robust and efficient compared to single-phase systems.

## 8. Pros and Cons of Biodigesters

### 8.1. Positive Impacts

**Efficient waste management:** Biodigesters offer an effective way to manage waste sustainably, transforming kitchen waste, agricultural residues, animal manure, and other organic materials into biogas and nutrient-rich digestate. This process reduces the amount of waste that ends up in landfills, thereby minimizing environmental pollution and greenhouse gas emissions [83].

**Renewable energy production:** Biodigesters generate biogas, a renewable energy primarily composed of methane and carbon dioxide, which can be used for cooking, heating, and electricity generation. By producing biogas, biodigesters enhance energy security, lower fossil fuel dependence, and contribute to climate change mitigation [83].

**Greenhouse gas emissions reduction:** During AD, biodigesters capture methane—a potent greenhouse gas—before it can escape into the atmosphere. This process helps reduce global warming, with biogas digesters estimated to prevent up to 0.25 °C of warming by the mid-century. When considering sectors like agriculture and mining, biogas offers a comparable impact in reducing methane emissions. In electricity generation, biogas can reduce global emissions by 18–20%, and over its lifecycle, it is 90% more effective at reducing greenhouse emissions than fossil fuels [15].

**Enhanced soil fertility:** The digestate produced by biodigesters is rich in nutrients such as nitrogen, phosphorus, and potassium, making it an excellent fertilizer. This by-product can improve soil fertility, boost crop yields, and support permaculture practices [83].

**Odor control and bacteria reduction:** Biodigesters help control the odors typically associated with organic waste decomposition, leading to a more pleasant environment. Additionally, the anaerobic process eliminates harmful bacteria, reducing the risk of water and soil contamination and benefiting public health [83].

**Pathogen elimination:** AD significantly reduces the pathogens in organic waste, making the resulting digestate safer for agricultural applications. A study from Poland demonstrated that a biodigester effectively eradicated pathogens like *Salmonella Senftenberg*, *Enterococcus* spp., and even deactivated *Ascaris suum* eggs during the co-digestion of food waste [5].

**Economic incentives:** Biodigesters offer financial opportunities, particularly for small farms, through carbon credits awarded for methane reduction. These credits can be sold to larger companies seeking to offset their emissions. Moreover, biogas production supports a circular economy by providing energy and chemical inputs necessary for farm operations [84].

**Support for sustainable agriculture:** Biodigesters promote sustainable agricultural practices by improving soil health, enhancing water management, minimizing pollution, and aiding in carbon sequestration. The bio-slurry produced is odorless, reducing nitrate contamination in water supplies, and the biogas generated can power farm operations, advancing overall resilience and biodiversity [85].

### 8.2. Negative Impacts

**Risk of leakage and contamination:** Biodigesters carry an environmental risk if methane leaks or waste is not adequately contained. Flaring of excess methane is required but often unmonitored; non-compliance can lead to environmental issues. For instance, an Ohio biodigester, Renergy, faced lawsuits in 2022 due to foul odors from insufficient flaring, with an estimated 1.5 million gallons of untreated waste at risk of leaking [86]. Biodigesters, while highly beneficial for sustainable food waste management and renewable energy production, require careful operation to mitigate these potential drawbacks. The accumulation of methane (CH_4_) gas in enclosed structures poses a significant explosion hazard, particularly in anaerobic digestion (AD) facilities, landfills, and industrial biogas plants. Methane is a highly flammable gas with a lower explosive limit (LEL) of 5% and an upper explosive limit (UEL) of 15% in air, meaning that even moderate concentrations in confined spaces can result in combustion or detonation upon ignition. The presence of oxygen leaks, static electricity, or mechanical sparks in poorly ventilated reactor chambers or storage areas further increases the risk of explosion. Preventive measures, including gas leak detection systems, proper ventilation, pressure relief valves, and controlled flare systems, are essential to mitigate the hazard and ensure safe biogas handling and storage [16].

**High feedstock requirements:** Biodigesters require significant amounts of organic material, leading some facilities to cultivate specific crops solely as energy feedstock. This practice can increase the demand for land, water, and greenhouse-gas-intensive agricultural practices necessary for crop production [86].

## 9. Case Studies

Biodigesters have been adopted globally (Table 4) [87,88,89]. Biodigesters in the United States have increasingly become integral to sustainable waste management and renewable energy generation (Table 5). In locations such as the Newtown Creek Facility in NYC, biodigesters process large amounts of organic waste, reducing environmental impacts while producing substantial energy. Facilities like Vanguard Renewables and Quantum Biopower contribute to regional waste solutions by handling diverse feedstocks, from food scraps to agricultural waste. California’s dairy digesters reflect the state’s commitment to methane capture and renewable gas production. Collectively, these projects demonstrate how biodigester technology can mitigate food waste challenges, offset fossil fuel dependence, and support cleaner energy alternatives across varied community and industrial settings.

These biodigesters demonstrate a range of applications and scales, from municipal waste handling in densely populated cities like Zhenjiang, China, to large-scale agricultural waste processing in Denmark. Facilities in places like Italy and the UK have shown how biodigesters can significantly reduce CO_2_ emissions and convert waste into renewable energy, supporting national sustainability goals. The diversity in technology—such as the portable systems in Chennai and the large centralized plants in Europe—illustrates the adaptability of biodigesters across different environments and waste management needs globally [102].

Additionally, reflecting on the case studies outlined in Table 5, several key practices for biodigester maintenance and improvement can be derived. First, regular monitoring and maintenance are crucial to prevent system failures. Implementing secondary control systems for emissions, such as hydrogen sulfide (H_2_S), can mitigate environmental risks and improve operational efficiency. Additionally, system reliability is paramount; the Newtown Creek Facility’s experience with flaring excess gas during National Grid outages highlights the need for backup systems to avoid environmental harm and community disruption. Economic sustainability is another key consideration. While the Newtown Creek Facility’s model of selling biogas and environmental credits under the EPA’s Renewable Fuel Standard Program is innovative, the short-term hike in local utility bills underscores the importance of balancing initial investments with long-term economic benefits [90,91,92]. Furthermore, cluster systems, as demonstrated in California’s dairy digester projects, can enhance scalability and efficiency by pooling resources from multiple farms [95,96].

In regard to the physical maintenance of the biodigester, the key practices include regular monitoring of feedstock quality and ensuring proper substrate mixing to maintain homogeneity, which directly impacts methane production. Temperature control is critical, as methanogenic bacteria are sensitive to fluctuations; thus, the heating system should be checked frequently to maintain optimal digester conditions. Avoiding the introduction of foreign compounds like soap or inhibitors is crucial, as these can disrupt microbial activity and reduce the gas yield. Regular inspection of gas pipelines, valves, and the inlet/outlet systems is necessary to prevent leaks and blockages, and to ensure smooth gas flow. Scum and siltation should be removed periodically to avoid hydraulic blockages and maintain efficient digester function. Additionally, adhering to the correct water-to-dung ratio is vital to prevent issues like thick bio-slurry overflow. If issues do arise, Table 6 describes the fixes for common problems with biodigesters [103].

While biodigesters provide many environmental and economic benefits, their broader adoption faces several challenges, including the high initial setup costs, the need for specialized technical skills, and issues with scaling. Infrastructure requirements, feedstock availability, digestate management, and gas storage are additional obstacles. Yet advancements in technology and ongoing research are likely to make biodigesters more feasible, affordable, and appealing worldwide in the coming years.

## 10. Key Challenges and Considerations

### 10.1. Operational Limitations

Although biodigesters are advantageous, they face operational challenges that can influence both efficiency and productivity. These include fluctuations in nutrient levels, changes in temperature and pH, potential microbial imbalances, and risks associated with gas leakage or system malfunctions. Proper design, regular maintenance, and continuous monitoring are essential to ensure that biodigesters operate effectively and produce stable biogas yields. A summary of the problems, causes, and remedies in a biogas plant is provided in Table 6 [103].

### 10.2. Feedstock Availability

Consistent and quality feedstock is essential for effective biogas production, but the availability can vary seasonally or be limited by logistical constraints. Factors such as access to local food processing waste, seasonal fluctuations in waste generation, and competition with other uses can impact feedstock supplies. Biodigester projects must assess local resources, plan for diverse feedstock sources, implement efficient storage and collection strategies, and explore co-digestion with various organic materials to overcome these challenges.

### 10.3. Economic Viability

Biodigester projects often require significant upfront capital and ongoing operational investments, making financial stability a key consideration. The costs associated with setup, maintenance, and operation, and the potential revenue from energy or fertilizer sales, play a critical role. Securing funding, exploring alternative financing options, and fostering public–private partnerships can help improve the economic outlook for biodigester projects. Enhancing system efficiency and optimizing energy output can further contribute to financial sustainability. Traditional food waste management methods pose a hefty cost. Haula, a national waste collection company, reports that a workweek’s worth of waste pickup (around 5 cubic yards of waste) costs around USD 2600. On average, as of 2023, a municipal solid waste landfill tipping is around USD 56.8 per ton, and in the Northeast specifically, around USD 83.44 per ton [104]. One study found that universities of around 20,000 students can spend up to USD 704,000 annually on food waste [105]. Regardless, large-scale disasters also require high capital investment. For a ton of feedstock, the capital costs range between USD 455 and USD 608 and the operating costs vary from USD 18 to USD 100. However, these systems also reduce costs by generating biogas and providing fertilizer (USD 34–122 savings per ton of fertilizer) [18]. On the other hand, biodigesters have high capital and installation costs, with a University of Kwazulu-Natal study reporting that the installation costs account for 28.5% of the total costs in biodigester-based food disposal. Regarding the capital costs, it has been reported that the Biogas-Pro biodigester’s cost itself is 59% of the total financial cost of the biodigester. However, there is high potential for a positive return, with the economic internal rate of return (IRR) reaching as high as 57.68% and the benefit–cost ratio (BCR) of 4.83. Many of the economic benefits of biodigesters arise from energy costs [106]. Nevertheless, biodigesters are typically not economically viable in rural settings. For instance, the Biogas-Pro biodigester used in rural South Africa cost USD 2857 and was installed for 4840 but boasted a benefit-cost-ratio of 0.98, suggesting that the revenue could not cover the costs of the device, even with the ZAR 180.57/month saved on fuels and ZAR 30.82/month saved on bio-slurry replacing fertilizers. On the other hand, urban implementations of biodigesters typically have higher economic benefits. The Nanning Biogas Taxi Project generated biogas from tapioca waste at a cost 50% cheaper than gasoline, saving taxi drivers USD 15/day on fuel and doubling their income. Other urban biogas initiatives, such as the East Bay Municipal Utility District in Oakland, Sadia’s Sustainable Swine Program, and Yanqing Deqingyuan Eco-Garden in Beijing rely on carbon credits and electricity sales for revenue [106].

### 10.4. Temperature Control

Effective substrate heating is crucial to optimizing biogas production, but achieving and maintaining consistent temperatures can be challenging. Fluctuations in temperature impact the biochemical reactions within anaerobic digesters, affecting the energy recovery and system efficiency. Solar-assisted biodigester systems (SABs), though promising, have high implementation costs and can be affected by weather variability, which can interrupt continuous energy supply. Advanced heating systems require significant capital and labor for installation and upkeep. In cold climate conditions, sustaining optimal anaerobic digestion (AD) reactor temperatures presents a considerable energy burden, as increased thermal inputs are required to maintain microbial activity. Anaerobic digesters typically operate within mesophilic (35–40 °C) or thermophilic (50–55 °C) ranges, necessitating continuous heating to counteract ambient temperature fluctuations. The thermal energy demand for biomass heating in such environments can constitute 30–50% of the total process energy consumption, thereby diminishing the overall net energy recovery. Insufficient heating leads to suboptimal microbial metabolism, prolonged hydraulic retention times (HRT), and decreased methane yield, ultimately impairing system efficiency.

### 10.5. Climate Justice

While there has been thorough research on the economic, health, and environmental benefits of biodigesters, certain existing research expresses concerns regarding the climate justice metric and the use of biodigesters. Specifically, there has been discussion on biogas primarily benefitting industrial agriculture while burdening rural and low-income communities with exposure to flare-ups and excess methane, ammonia, hydrogen sulfide, etc [107]. Others critique biogas projects for redirecting funding from sustainable agricultural practices, thereby damaging farmers in the short term. To elaborate, the capital costs for a manure digester oftentimes exceed USD 1 million and have a payback period of 5–6 years, thereby not serving as a financially feasible option for many farmers. Additionally, others have critiqued states such as California for directing USD 200 million of its climate investment funds to digesters alone, instead of supporting sustainable agriculture [108].

### 10.6. Global Expansion and Adoption

As industrialization and waste production increase worldwide, there is a growing need for sustainable waste management practices. Many developed countries are exploring circular economy principles, which aim to extend the life cycle of products and materials to minimize waste. However, less emphasis has been placed on adapting these practices to benefit low- and middle-income countries. A shift from a linear “take-make-dispose” economy to a circular model is necessary to sustain resources for future generations, enabling countries to recycle materials, reduce reliance on raw resource extraction, and mitigate environmental impacts.

In developed regions, adopting circular economy practices has shown promising economic benefits, with the European Union estimating that it could generate up to EUR 600 billion by implementing circular economy strategies in the manufacturing sector alone. China has also adopted measures focused on improving resource efficiency, notably implementing a ban on plastic waste imports in 2018, which redirected waste disposal efforts back to Western nations. In contrast, in lower-income nations, the lack of waste management infrastructure has resulted in practices like open dumping, which leads to severe soil and water pollution. Countries dealing with rapid urbanization often lack sustainable disposal systems, leading to heightened environmental and public health concerns. Implementing biodigester technology could transform waste management in these regions by generating renewable energy and providing organic fertilizer, thus reducing dependency on non-renewable resources. A study conducted in South Africa examining biodigester adoption among farmers highlighted factors that influence uptake, including awareness of environmental issues and knowledge of biodigester benefits. Younger and more literate populations showed greater interest in adopting biodigester technology, while limited understanding of the environmental damage caused by conventional fertilizers and waste accumulation posed a barrier. Educating communities about biodigesters and their benefits, as well as providing financial incentives, will be essential for global adoption.

In summary, while biodigesters offer a sustainable approach to waste management, addressing economic, technical, and awareness challenges will be essential for widespread adoption. A concerted global effort is required to promote education and innovation, supporting communities worldwide in embracing biodigesters as an alternative to traditional waste management practices. By leveraging biodigesters, societies can transition toward a circular economy that conserves resources, reduces emissions, and mitigates environmental harm.

## 11. Future Prospects and Research Areas

Continued research on biodigesters is needed to tackle the chronic problems faced by society that are exacerbated by growing populations, larger accumulations of waste, economic needs, and climate change. This will require newer models, multiple options for the digestion process, and approaches that consider the waste before, during, and after the digestion is completed. In the future, this industry will not only need to develop its own products and system but also integrate into existing systems and be adjusted to handle many environments, which include but are not limited to wastewater treatment plants and densely populated neighborhoods.

Research into biodigesters is needed not only to improve the model and procedure but also to develop guidelines and regulations for this emerging industry. With limited regulations comes room for reckless measures that can cause preventable harm. Policymaking is necessary to outline the general process and expectations for taking the current waste to landfill mentality and promoting the waste to re-use method to reduce greenhouse gas emissions [109].

Research into hybrid digesters is becoming more prominent as the conventional three types of biodigesters are not sufficient to address all environments and conditions. By combining specific aspects of each style of biodigester, a new hybrid model can be tested to determine if it is able to address a particular need, as referenced in a previous study on constructing wetland habitats [82].

There is a growing urge to replace fossil fuels with biomethane and AD has become the center of the conversation for companies seeking to reach their goals of cleaner energy. Another factor driving the increase in AD projects is legislation and policies that require food waste not to be dumped into landfills. “Methane poses a greater climate risk than carbon dioxide as it has about 281 times the global warming potential as carbon dioxide over 20 years”. With such a staggering statistic, the methane release when organic matter degrades must be reduced. Food is one of the largest contributors in terms of waste that enters landfills, which is a concern that can be addressed by additional AD plants. As more legislation and guidelines are passed in the US and Europe, more companies will develop to meet the current unmet needs in the methane production industry, looking to capitalize on this emerging market as fast as needed technology, pretreatments, and software are developed.

### 11.1. Improving Biodigester Efficiency

Research strategies to improve biodigester efficiency rely on optimizing the conditions under which the digester operates, encouraging growth among the communities of microorganisms, and finding the most practical feedstock to apply. To do this, monitoring systems are needed to track the changes in the digestion process, design improvements should be tailored to the environment, and a more digitized operational system is necessary to streamline the process. While biodigester efficiency is focused on improving the process, it is also important that it is able to improve the environment it occupies and make a difference in the lives of the people who are utilizing the biodigester. Since the goal of sustainability is to reduce waste and elongate the life cycle of products, the intention for the biodigester is to manage the waste overload and alleviate the burden developing countries have been dealt when it comes to receiving other countries’ wastes.

### 11.2. Development of Biogas Purification Technologies

Multiple technologies and processes are necessary to digest the particular feedstock and organic matter combined in the digester. The current processes include extraction, biohydrogen production, gasification, and hydrolysis [20]. While these technologies have proven to be a strong start to handling the waste and producing digestate, future technologies will need to be developed to better produce digestate more efficiently and deal with post-digestion matter to extract and separate the nutrients in each composition.

Maximizing the biogas generated is possible by controlling parameters that affect the activity of the microbes and movement in the digester. These factors include the hydraulic retention time (HRT), temperature, pH, C/N ratio, and seeding, to list a few [20]. Since the feedstock must be optimal for the region of the climate to allow for the most favorable conditions for microbes to perform, new feedstock is consistently needed to adapt to the various environments globally. For newer technologies, the ability to prevent hazardous by-products that are also generated by biogas, like H_2_S, Si, Co, and NH_3_, is required [110]. Other challenges include purifying biogas, which would require post-treatment to remove impurities after biogas production, or an alternative treatment that can produce biogas without H_2_S, which has the most negative concerns compared to the other gases mentioned. Overall, the goal of identifying new purification technologies is enhancing the current progress to digest waste more efficiently, as this will lead to quicker turnaround times or better handling of the post-digestion material.

### 11.3. Exploring Novel Feedstock Sources

Some of the current feedstocks include using oil-based residues like animal fats and used cooking oils; organic wastes like manure, municipal solid waste, or sewage solids; agricultural residues like corn cobs, nut shells, and crop residues; and forest residues like bark, leaves, pulpwood, and sawdust [111]. The search for new feed sources is critical to expanding the reach biodigesters have globally. This can only be achieved experimentally by mixing current feed sources while also testing a variety of plants, wastes, and other crops that grow in the environment the biodigester is placed in. Otherwise, without progress, the lack of feed sources will serve as an impediment to this growing industry.

## 12. Summary and Conclusions

The rise in the use of biodigesters has been facilitated by a drive for environmentally sustainable methods of waste management, and AD has already demonstrated its promise as a technology with a diverse range of applications, from food waste and agriculture to wastewater treatment. The operational mechanisms of food waste biodigesters were investigated to describe the commonly available systems and a list of successful food waste biodigesters in the US as well as in the rest of the world was provided. The key challenges of the operation of food waste biodigesters were listed to describe the much-needed improvements for food waste biodigesters. While a great deal of understanding of the underlying science has been accumulated and many innovations in terms of optimization were proposed, there still exist gaps that impede researchers from having a complete understanding of the intricate process that underlies food waste biodigesters. Factors pertaining to the design, pretreatment methods, and digester conditions of food waste biodigesters should all be considered to ensure the design of efficient and cost-effective food waste biodigesters.

## Figures and Tables

**Table 1:** Quantitative assessment of the microbial contamination, health risks, and environmental impacts of food waste.

Category	Factor	Measurements and Units	Health Risks	Ref.
Bacterial Contamination	*Salmonella* spp.	10^3^–10^6^ CFU/g (colony-forming units per gram) in landfills	Severe gastroenteritis, dehydration, systemic infection in immunocompromised individuals; 1.35 × 10^6^ cases/year, 420 deaths (~0.0004% of US population).	[28]
Bacterial Contamination	*E. coli*	10^2^–10^5^ CFU/g in landfill samples	Hemorrhagic colitis, kidney failure (hemolytic uremic syndrome—HUS), severe diarrhea; 2.65 × 10^5^ cases/year, 100 deaths US population.	[29]
Bacterial Contamination	*Listeria* *monocytogenes*	10^4^–10^7^ CFU/g in landfill samples	Meningitis, sepsis, stillbirth in pregnant women, high mortality in vulnerable populations; 1.6 × 10^3^ cases/year, 260 deaths US population.	[30]
Food Waste	Annual Food Waste (US and Global)	US: 61% of food wasted (~60 M metric tons) Global: >1B metric tons	Increased vector-borne diseases such as rat infestations, bacterial proliferation, and air pollution-related illnesses.	[31]
Green House Gas (GHG) Emissions	Methane (CH_4_) Emissions from Landfills	34 metric tons CH_4_ per 1000 metric tons of waste; CH_4_ is 281 × more potent than CO_2_	Respiratory diseases, cardiovascular stress, and long-term exposure linked to neurotoxicity.	[32]
VOC Emissions	Mixture of VOCs	Ethanol: 2–10 mg/m^3^; ammonia (NH_3_): 5–50 mg/m^3^; hydrogen sulfide (H_2_S): 1–10 ppm	Irritation of eyes, nose, and throat; long-term exposure linked to liver, kidney, and nervous system damage.	[33]

**Table 2:** Health hazards of disease vectors attracted to food waste in landfills.

Disease Vector	Description	Health Risks to Humans	Conditions Favoring Infestation
Rodents (Rats)	Common disease carriers attracted to large amounts of food waste in landfills. They can transmit diseases through bites, urine, fleas, and feces.	Hantavirus, leptospirosis (both can be fatal).	High availability of food and water, favorable burrowing sites, such as sewers and underground subways in urban areas.
Flies (Houseflies, Blowflies)	Attracted to decaying food and organic waste in landfills. They feed on rotting material and then move to other surfaces, including humans, carrying bacteria.	Shigella virus (can cause dysentery and diarrheal diseases).	Presence of decaying food waste, suitable for breeding and feeding.
Mosquitoes	Accumulation of food waste creates stagnant water pools, attracting mosquitoes. They are efficient disease vectors, transmitting pathogens through bites.	Malaria, dengue, zika, West Nile virus, chikungunya	Stagnant water and organic waste, high organic decomposition rates; rapid mosquito population growth in warm, humid conditions.

**Table 3:** Comparative table of biodigesters.

	Fixed-Dome Plant	Floating-Drum Plant	Balloon Plant
Lifespan	20 years or more	15 years maximum	2–5 years
Size	5–200 m^3^	5–15 m^3^	4–100 m^3^
Investment Costs	Low	High	Low
Cost of Maintenance	Low: since they have no moving parts	High: require regular painting and de-rusting	Low
Regional Suitability	Suitable for rural areas: ready availability of materials such as bricks and cement	Not suitable for coastal areas; suitable for remote or mountainous areas	Suitable for remote or mountainous areas
Climate Suitability	Resistant to temperature fluctuations	Not suitable in high-humidity or corrosive environment	Highly sensitive to ambient temperature
Gas Pressure (mbar)	60–130	20–30	Low
Gas Pressure Control	Medium: lack of an expansion chamber	High: constant gas pressure due to 8–10 cm water column	Low: excessive pressure can cause damage
Skill Required	High	High	Low
Methane Emissions	High	Medium	Low
Scum Formation Potential	Possible: lack of mechanical agitation, especially in larger plants	Possible: stirring mechanisms required for prevention	Unlikely: natural agitation
Types	BORDA model: combines fixed-dome and floating-drum designs for constant gas pressure CAMARTEC model: features a weak ring to prevent cracking in the dome DEENBANDHU model: low-cost design using brick and cement, with a newer version using woven bamboo. JANATA model: brick-reinforced fixed-dome structure with integrated gas holder and digester	KVIC model: cylindrical digester with a floating drum PRAGATI model: hemisphere-shaped digester GANESH model: made of angular steel and plastic foil	Channel-type digester: covered with sheeting or a sunshade

**Table 4:** Global case studies.

Region	Project Name	Initial Capital Investment	Operational and Maintenance Costs	Feedstock Availability	Biogas and Digestate Sales Revenue	Energy Yield	Environmental Benefits	Policy Incentives	Payback Period
China	Beijing Fangshan District Doudian Village Central Biogas Supply System	USD 1 million	Not specified	44 tons of cow dung/day (~1000 cows)	Biogas sold to 1900 households at 30 US cents/m^3^; effluent sold as organic fertilizer	2000 m^3^ of methane/day	Reduces reliance on LPG and burning of wheat straw	Aligns with national clean energy goals	Not specified
	Beijing Yanqing Deqingyuan Eco-Garden 2 Trillion Watts Poultry Manure Biogas	USD 10 million	Not specified	212 tons of chicken manure/day	Biogas-generated power sold to the grid; excess biogas sold as cooking gas; effluent used as fertilizer	7 million m^3^ of biogas/year, generating trillion watts of power	Annual greenhouse gas reduction of 80,000 tons of CO_2_ equivalent	Recognized as a UNDP/GEF large-scale biogas-power demonstration project	Not specified
	Anhui Fuyang Jingwei Agro Recycling Co. Integrated Biogas Project	USD 170,000	Not specified	Pig manure	Biogas used for electricity generation; effluent used in 800 vegetable greenhouses	60 kW of electricity	Reduces pollution from untreated animal waste	Aligns with circular agriculture principles	Annual savings of USD 30,000, suggesting a payback period of ~5–6 years
	Nanning Anning Starch Co. Purified Biogas as Car Biofuel	USD 6 million	Not specified	1000 tons/day of wastewater from tapioca alcohol manufacturing	Biogas purified and sold as car biofuel; treated wastewater provided free to local farmers	30,000 m^3^/day of biogas, purified to 21,000 m^3^/day of biofuel	Annual CO reduction of 20% and CO_2_ reduction of 99%	Supported by the Nanning government	Annual revenue of USD 4 million suggests a payback period of ~1.5 years
Brazil	Born Despacho Farm, Minas Gerais (Clean Development Mechanism)	Unknown (paid by AgCert)	Long-term maintenance issues	Pig manure from 500 pigs	Biogas used for electricity generation; effluent used as high-grade fertilizer	12,500 m^3^ of biogas/day	Reduces greenhouse gas emissions and improves animal waste management	Part of a clean development mechanism (CDM) project	Not specified
	Sadia’s Sustainable Swine Production Program (3S Program)	Not specified	Not specified	Pig manure from Sadia’s swine suppliers	Biogas used for on-site electricity generation; carbon credits sold	Not specified	Reduces greenhouse gas emissions from over 3500 swine producers	Part of Sadia’s Sustainable Swine Production Program (3S Program)	5-year payback time
	Cabra Forte (Strong Goat)—Bahia, Brazil (USAID Project)	USD 700 per biodigester	Not specified	Goat manure	Biogas used for lighting and cooking; effluent used as organic fertilizer	Not specified	Improves waste management and reduces reliance on traditional fuels	Supported by USAID and local banks	Not specified
Central America	Costa Rica, Sante Fe de Guatuso (Polyethylene Tube Biodigester)	USD 300 per biodigester	Not specified	Cow and pig manure from local farms	Biogas used for cooking stoves	Not specified	Reduces reliance on wood burning, improving air quality and reducing deforestation	Supported by the UN Women’s Group and local agricultural ministries	Not specified
	Costa Rica, Alajuela (Dos Pinos Dairy Farm Integrated System)	USD 19,275	Not specified	Manure from 80 cows	Biogas used for electricity generation; effluent and compost sold as byproducts	100 tons of electricity	Reduces waste and provides renewable energy	Supported by local environmental agriculture programs	Not specified
	Costa Rica, Guapiles (Chiquita Brands Biodigester)	Not specified	Not specified	Food waste from fruit processing	Biogas used for energy in the manufacturing plant; effluent used as fertilizer for local farmers	Not specified	Reduces carbon emissions and provides sustainable energy	Part of Chiquita’s corporate sustainability initiative	Not specified
	Honduras, El Progreso (Palm Oil Mill Biodigester)	Not specified	Not specified	Wastewater from palm oil processing	Biogas used for electricity generation; effluent used as irrigation water	300 m^3^ of biogas/hour	Reduces methane emissions and treats wastewater	Part of a CDM project	Net present value of USD 542,299 over 10 years
India	Pune, South India (ARTI Balcony Biogas Plant)	USD 200 per biodigester	Not specified	Household food waste	Biogas used for cooking and heating; effluent reused in the biodigester	Reduces LPG use by half	Reduces CO_2_ emissions by 0.3–0.6 tons/year	No government subsidies, but the system is affordable	2 years, based on savings from LPG
	Shirdi, Maharashtra (Sulabh Toilet-to-Biogas System)	USD 2000 for biogas plant attachment	Not specified	Human waste and wastewater	Biogas used for electricity and cooking; effluent used as fertilizer	Electrifies the site	Reduces public health hazards and provides renewable energy	Supported by Sulabh International	Not specified
	Kerala, South India (Biotech Institutional Biodigester)	USD 220 (with 60% government subsidy)	Not specified	Food and organic waste	Biogas used for cooking and electricity; effluent used as fertilizer	3 kW of electricity for institutional use	Reduces organic waste and provides renewable energy	Government subsidies available	3 years, based on savings from LPG
United States	Milwaukee, WI (Growing Power Urban Farm)	Not specified	Not specified	Agricultural wastes and food residues	Biogas used for heating and electricity	Handles 5 tons of food waste/day	Reduces waste and provides renewable energy	Part of a community economic development model	Not specified
	Oakland, CA (East Bay Municipal Utility District Food Waste Co-Digestion)	Not specified	Not specified	Food waste from restaurants and grocery stores	Biogas used for electricity generation	Increased electricity production at wastewater treatment facilities	Reduces food waste and provides renewable energy	Supported by the US EPA and local utility districts	As little as 3 years, depending on existing infrastructure

**Table 5:** Overview of anaerobic digester facilities in the United States.

Location	Established	Description	Key Learnings and Challenges	Ref.
Newtown Creek Facility, Brooklyn, NY, USA	February 2024	185-foot-high anaerobic digester at wastewater treatment plant; supports curbside composting in NYC with 250 smart bins.	Economic model: Customers pay a competitive per-ton fee for waste disposal. The project also sells biogas and environmental attribute credits under the EPA’s Renewable Fuel Standard Program to heat over 5000 homes by routing excess biogas to the National Grid. Local utility bills have observed a hike to compensate for the initial investment. System reliability: When the National Grid system is down, excess gas must be flared, harming the community and the environment.	[90,91,92]
Vanguard Renewables	Various	Projects at various farms such as Goodrich Family Farms, VT, Spencer, MA). Rutland farm processes 20,000 tons of food waste and 9125 tons of manure annually, producing 4200 MWh of energy.	H_2_S emissions: Lack of a secondary control system for hydrogen sulfide emissions led to excess emissions. Digester failure: Nov 2021 and March 2022 failures released 51,309 cubic feet of biogas and 1.3 million gallons of digestate.	[93,94]
California	Various	227 dairy digester projects, capturing methane from 255 dairy farms. Produces renewable electricity, natural gas, or hydrogen fuel.	Cluster system: The use of a biogas facility cluster system. Hydrogen use: Use hydrogen from dairy biomethane to fuel trucks and cranes at the LA Port.	[95,96]
Jessup, MD	Unknown	Processes up to 125,000 tons/year of organic by-products and wastewater from food industry. Produces biomethane for 4800 homes.	Regulations: State laws are driving large food generators such as supermarkets, cafeterias, food producers to divert food waste from landfills to digesters. Slow roll-out of law. Location: Digester located in the Maryland Food Center, where 100,000 tons of food waste	[97,98]
Central Vermont Recovered Biomass Facility	Unknown	Daily feedstock mixture of 10 tons of dairy manure and 15 tons of food scraps powers a 250 kWh co-generation plant, producing up to 2 million kWh annually.	Education: The facility is focused on education and research in renewable energy, waste management, and sustainable agriculture at the Vermont Technical College. Economy: Governor emphasized the project’s role in creating jobs	[99,100]
Flood Brothers Farm, Maine	Unknown	Biodigester operating on cow manure producing 130,000 dekatherms of natural gas per year, covering 40% of Summit Natural Gas’s residential load.	Economy: Redistributing excess manure to farmers as fertilizer.	[101]

**Table 6:** A summary of the problems, causes, and remedies in a biogas plant from.

Problems	Causes	Remedy Measures
Reduction in the gas yield	Reduced performance in terms of methanogenic bacteria production	Proper and quality substrate mixing
	Reduction in feedstock quality	Check the heating system
	Temperature drops in the biodigester	Check the level of potential inhibitor compounds
	Inhibition by foreign compounds	Add bio-slurry from a foreign digester if the methanogenic bacteria have dropped
	Non-homogenous substrates	
Methane concentration drop	Reduced feedstock quality	Proper and quality substrate mixing
	Temperature drops	Check the heating system in the biodigester
	Inhibition by foreign compounds	Check the composition of the inhibitor compounds in the feedstock
Foaming problem	A new substrate with high protein content has been added	Reduce or stop feeding the biodigester
	Air is introduced in the digestion chamber	Analyze feedstock concentration
	Temperature changes in the biodigester	Reduce the introduction of air in the biodigester
pH drops in the biodigester	Abnormal feeding rate in the biodigester	Reduce the input in the biogas plant until the system returns to normal
	Change in operating temperatures	Use only manure until the system returns to normal
	Introduction of foreign compounds like soap	Check thoroughly what is entering the biodigester
	Inhibition by foreign compounds	Analyze the chemical composition of the feedstock and the input entering the biodigester
Reduction in gas volume	Underfeeding of the plant	Follow the feeding instructions
Insufficient gas pressure and maximum pressure not reached	Gas leaks	Check for gas leaks
	Scum formation in the digester	Check the biodigester climate/constituents
	The improper mixture ratio of water and cow dung	Follow normal mixing procedure preferably of the ratio 1:1. or 1:0.75; do not make the mixture too thin or too compact
	Siltation in the digester	
Bio-effluent smelling at the expansion chamber and the canal.	Overfeeding of the digester	Follow feeding instructions
	Leakage in the biodigester	Check for gas leaks
Gas stove not burning well	Blocked primary ducts	Clean the air ducts and burner holes
	Blocked flame holes	Adjust the primary air knob to get the correct mixture
	Incorrect gas mixture ratio	Open the water drain valve to remove accumulated water in the pipe
The lamp is not bright enough	Dirty glass screen	Clean the lamp glass screen
	Cracked, blockage, and destroyed mantle	Replace the cracked mantle
	Presence of water in the system	Adjust the primary air knob to get the right mixture
		Clean the mantle holder hole
		Enforce traps along the piping lines
Low gas quantity in the biodigester	Underfeeding/irregular feeding of the biodigester	Follow feeding instructions as per the logbook
	Scum formation in the biodigester	Proper mixing and removal of impurities
		Remove the scum
No gas reaching the appliances	Blocked water passage in the pipeline system	Check for the presence of water
	No gas/insufficient gas is being produced in the digester	Check for leaks
	Disconnected gas pipeline	Check for pipe disconnection and connect it back
	Impurities in the digester	Check the main gas valve
	Closed main gas valve at the test unit chamber	
The feeding material does not enter into the digester	Blocked inlet pipe	Poke through the inlet pipe
	The position of the inlet pipe is below the overflow point	Ensure right vertical dimensions are used
		Take out the inlet pipe for inspection
Thick bio-slurry overflow	Incorrect water/dung mixing ratio	Implement a good mixing ratio
	No hydraulic movement in the digester	Check for water leakages
	Water leakage in the digester	Ensure daily use of the gas to allow hydraulic movement
	Water leakage in the expansion chamber	Check for cracks in the digester and the mixing and expansion chamber
Slurry entering the gas pipe	Gas outlet pipe placed below the overflow point	Check slurry overflow point
		Reduce slurry overflow point to lower level
		Put a filter to allow only gas to pass through, blocking the slurry in the process

## Data Availability

No new data were created or analyzed in this study.

## References

[1] Igini M. (2022). 10 Food Waste Statistics in America. https://earth.org/food-waste-in-america/#:~:text=Food%20goes%20to%20waste%20at,66%20billion%20pounds)%20every%20year.

[2] WWF (2021). Driven to Waste: The Global Impact of Food Loss and Waste on Farms Publications| WWF. World Wildlife Fund. https://www.worldwildlife.org/publications/driven-to-waste-the-global-impact-of-food-loss-and-waste-on-farms.

[3] RTS (2024). Food Waste in America in 2024: Statistics & Facts. Recycle Track Systems. https://www.rts.com/resources/guides/food-waste-america/.

[4] Buzby J. (2022). Food Waste and Its Links to Greenhouse Gases and Climate Change. Usda.gov. https://www.usda.gov/about-usda/news/blog/2022/01/24/food-waste-and-its-links-greenhouse-gases-and-climate-change.

[5] Manyi-Loh C., Mamphweli S., Meyer E., Okoh A., Makaka G., Simon M. (2013). Microbial Anaerobic Digestion (Bio-Digesters) as an Approach to the Decontamination of Animal Wastes in Pollution Control and the Generation of Renewable Energy. Int. J. Environ. Res. Public Health.

[6] CDC Food Outbreak. https://www.cdc.gov/foodborne-outbreaks/index.html.

[7] Ricci A., Allende A., Bolton D., Chemaly M., Davies R., Fernández Escámez P.S., Girones R., Herman L., Koutsoumanis K., EFSA Panel on Biological Hazards (BIOHAZ) (2018). Listeria monocytogenes contamination of ready-to-eat foods and the risk for human health in the EU. EFSA J..

[8] Luisa B.G. (2012). Making Safe Food: A Management Guide for Microbiological Quality.

[9] de la Caba K., Guerrero P., Trung T.S., Cruz-Romero M., Kerry J.P., Fluhr J., Maurer M., Kruijssen F., Albalat A., Bunting S. (2019). From seafood waste to active seafood packaging: An emerging opportunity of the circular economy. J. Clean. Prod..

[10] Kliemann N., Al Nahas A., Vamos E.P., Touvier M., Kesse-Guyot E., Gunter M.J., Millett C., Huybrechts I. (2022). Ultra-processed foods and cancer risk: From global food systems to individual exposures and mechanisms. Br. J. Cancer.

[11] Anastasiou K., Baker P., Hadjikakou M., Hendrie G.A., Lawrence M. (2022). A conceptual framework for understanding the environmental impacts of ultra-processed foods and implications for sustainable food systems. J. Clean. Prod..

[12] Kadariya J., Smith T.C., Thapaliya D. (2014). Staphylococcus aureus and staphylococcal food-borne disease: An ongoing challenge in public health. BioMed Res. Int..

[13] Bryan F.L. (1978). Factors that contribute to outbreaks of foodborne disease. J. Food Prot..

[14] Lima Tribst A.A., de Souza Sant’Ana A., de Massaguer P.R. (2009). Microbiological quality and safety of fruit juices—Past, present and future perspectives. Crit. Rev. Microbiol..

[15] Kereng M.C. (2016). Diversity Profiling and Rapid Detection of Spoilage Yeasts in a Typical Fruit Juice Bottling Factory.

[16] Hsu S.-C., Chen H.-L., Chou C.-F., Liu W.-C., Wu C.-T. (2023). Characterization of microbial contamination of retail washed and unwashed shell eggs in Taiwan. Food Control.

[17] Rajendran K., Aslanzadeh S., Taherzadeh M.J. (2012). Household biogas digesters—A review. Energies.

[18] Haulla. https://www.haulla.com/blog/how-much-does-commercial-waste-collection-cost.

[19] Meegoda J.N., De Souza B.B. (2020). Briefing: Sustainable management of municipal solid waste without food waste. J. Environ. Eng. Sci..

[20] Meegoda J., Li B., Patel K., Wang L. (2018). A Review of the Processes, Parameters, and Optimization of Anaerobic Digestion. Int. J. Environ. Res. Public Health.

[21] Ishaq M., Ishaq H. (2022). Performance assessment of biogas-fed solid oxide fuel cell system for municipal solid waste treatment. J. Clean. Prod..

[22] Abad V., Avila R., Vicent T., Font X. (2019). Promoting circular economy in the surroundings of an organic fraction of municipal solid waste AD treatment plant: Biogas production impact and economic factors. Bioresour. Technol..

[23] Bong C.P.C., Ho W.S., Hashim H., Lim J.S., Ho C.S., Tan W.S.P., Lee C.T. (2017). Review on the renewable energy and solid waste management policies towards biogas development in Malaysia. Renew. Sustain. Energy Rev..

[24] Morero B., Vicentin R., Campanella E.A. (2017). Assessment of biogas production in Argentina from co-digestion of sludge and municipal solid waste. Waste Manag..

[25] Ferdeș M., Zăbavă B.Ș., Paraschiv G., Ionescu M., Dincă M.N., Moiceanu G. (2022). Food Waste Management for Biogas Production in the Context of Sustainable Development. Energies.

[26] Thakali A., MacRae J.D. (2021). A review of chemical and microbial contamination in food: What are the threats to a circular food system?. Environ. Res..

[27] Karanth S., Feng S., Patra D., Pradhan A.K. (2023). Linking microbial contamination to food spoilage and food waste: The role of smart packaging, spoilage risk assessments, and date labeling. Front. Microbiol..

[28] Centers for Disease Control and Prevention (CDC) (2024). About Food Safety. Food Safety. https://www.cdc.gov/food-safety/about/?CDC_AAref_Val=https://www.cdc.gov/foodsafety/foodborne-germs.html.

[29] Food and Drug Administration (FDA) (2019). *Escherichia coli* (*E. coli*). U.S. Food and Drug Administration. https://www.fda.gov/food/foodborne-pathogens/escherichia-coli-e-coli.

[30] Centers for Disease Control and Prevention (CDC) (2024). About Listeria Infection. Listeria Infection (Listeriosis). https://www.cdc.gov/listeria/about/index.html.

[31] Food and Agricultural Organization (FAO) (2021). Food Loss and Waste Database. Food and Agriculture Organization of the United Nations. https://www.fao.org/platform-food-loss-waste/flw-data/en/.

[32] United States Environmental Protectation Agency (USEPA) (2019). Basic Information About Landfill Gas. US EPA. United States Environmental Protection Agency. https://www.epa.gov/lmop/basic-information-about-landfill-gas.

[33] Boušková A., Dohányos M., Schmidt J.E., Angelidaki I. (2005). Strategies for changing temperature from mesophilic to thermophilic conditions in anaerobic CSTR reactors treating sewage sludge. Water Res..

[34] Espinoza R. (2020). How Does Leachate Contaminate Our Water Supply?. https://blog.idrenvironmental.com/how-does-leachate-contaminate-our-water-supply.

[35] Parvin F., Tareq S.M. (2021). Impact of landfill leachate contamination on surface and groundwater of Bangladesh: A systematic review and possible public health risks assessment. Appl. Water Sci..

[36] Rudziak P., Batung E., Luginaah I. (2024). The effects of gases from food waste on human health: A systematic review. PLoS ONE.

[37] Edwards C. (2024). The World Wastes More Than 1 Billion Meals Every Day as Hundreds of Millions Go Hungry, UN Report Finds. CNN. https://edition.cnn.com/2024/03/27/climate/un-food-waste-one-billion-meals-intl/index.html.

[38] Wang Z., Liang Z., Li G., An T. (2023). Odorous organic gas emission characteristics from cooked food wastes during aerobic decomposition. J. Clean. Prod..

[39] Oh H.Y.P., Humaidi M., Chan Q.Y., Yap G., Ang K.Y., Tan J., Ng L.C., Mailepessov D. (2022). Association of rodents with man-made infrastructures and food waste in Urban Singapore. Infect. Ecol. Epidemiol..

[40] Levine O.S., Levine M.M. (1991). Houseflies (Musca domestica) as mechanical vectors of shigellosis. Rev. Infect. Dis..

[41] Muhammad Nasir I., Mohd Ghazi T.I., Omar R. (2012). Production of biogas from solid organic wastes through AD: A review. Appl. Microbiol. Biotechnol..

[42] Alibardi L., Cossu R. (2015). Composition variability of the organic fraction of municipal solid waste and effects on hydrogen and methane production potentials. Waste Manag..

[43] Cusworth D.H., Duren R.M., Ayasse A.K., Jiorle R., Howell K., Aubrey A., Green R.O., Eastwood M.L., Chapman J.W., Thorpe A.K. (2024). Quantifying methane emissions from United States landfills. Science.

[44] Mills R. (2023). Waste Methane 101: Driving Emissions Reductions from Landfills. RMI. https://rmi.org/waste-methane-101-driving-emissions-reductions-from-landfills/.

[45] Abbasi T., Tauseef S.M., Abbasi S.A. (2012). Anaerobic digestion for global warming control and energy generation—An overview. Renew. Sustain. Energy Rev..

[46] Max K., Shannon K., Jenny S. (2023). Quantifying Methane Emissions from Landfilled Food Waste.

[47] US Environmental Protection Agency (2009). Nutrient Control Design Manual: State of Technology Review Report.

[48] Zhou L., Hülsemann B., Cui Z., Merkle W., Sponagel C., Zhou Y., Guo J., Dong R., Müller J., Oechsner H. (2021). Operating Performance of Full-Scale Agricultural Biogas Plants in Germany and China: Results of a Year-Round Monitoring Program. Appl. Sci..

[49] Palma A., Matamoros G., Escobar D., Sánchez A.L., Fontecha G. (2020). Absence of mutations associated with resistance to benzimidazole in the beta-tubulin gene of Ascaris suum. Rev. Da Soc. Bras. De Med. Trop..

[50] Biogas, a Climate and Clean Air Solution with Many Benefits. Climate & Clean Air Coalition. https://www.ccacoalition.org/news/biogas-climate-and-clean-air-solution-many-benefits.

[51] Czekała W. (2022). Digestate as a source of nutrients: Nitrogen and its fractions. Water.

[52] Bahgat E., Nasr F.A., Abuarab M.E., Haroun B., Hawash H.B., Liu R., Ibrahim M.M., Shana A., El-Qelish M. (2025). Unveiling graphite coated with nano-nickel ferrite for two-stage gaseous biofuel generation from potato processing wastewater. Biomass Bioenergy.

[53] Finstein M.S., Miller F.C., Hogan J.A., Strom P.F. (1987). Analysis of EPA guidance on composting sludge. Part I. Biological heat generation and temperature. BioCycle.

[54] Verma S. (2002). AD of biodegradable organics in municipal solid wastes. Columbia Univ..

[55] Liu C., Wang W., Anwar N., Ma Z., Liu G., Zhang R. (2017). Effect of Organic Loading Rate on Anaerobic Digestion of Food Waste under Mesophilic and Thermophilic Conditions. Energy Fuels.

[56] Markphan W., Mamimin C., Suksong W., Prasertsan P., O.-Thong S. (2020). Comparative assessment of single-stage and two-stage anaerobic digestion for biogas production from high moisture municipal solid waste. PeerJ.

[57] Pasteris A.M., Heiermann M., Theuerl S., Plogsties V., Jost C., Prochnow A., Herrmann C. (2022). Multi-advantageous sorghum as feedstock for biogas production: A comparison between single-stage and two-stage anaerobic digestion systems. J. Clean. Prod..

[58] Suksong W., Kongjan P., Prasertsan P., Imai T., O.-Thong S. (2016). Optimization and microbial community analysis for production of biogas from solid waste residues of palm oil mill industry by solid-state anaerobic digestion. Bioresour. Technol..

[59] Ajah C.P., Nwaojei K. (2024). Optimizing the microbial community composition in anaerobic digesters to improve biogas yields from food waste. World J. Adv. Res. Rev..

[60] Wang Z., Hu Y., Wang S., Wu G., Zhan X. (2023). A critical review on dry anaerobic digestion of organic waste: Characteristics, operational conditions, and improvement strategies. Renew. Sustain. Energy Rev..

[61] Jiang Y., Dennehy C., Lawlor P.G., Hu Z., McCabe M., Cormican P., Zhan X., Gardiner G.E. (2018). Inhibition of volatile fatty acids on methane production kinetics during dry co-digestion of food waste and pig manure. Waste Manag..

[62] Chew K.R., Leong H.Y., Khoo K.S., Vo D.V.N., Anjum H., Chang C.K., Show P.L. (2021). Effects of AD of food waste on biogas production and environmental impacts: A review. Environ. Chem. Lett..

[63] Holm-Nielsen J.B., Al Seadi T., Oleskowicz-Popiel P. (2009). The future of AD and biogas utilization. Bioresour. Technol..

[64] Persson M., Jönsson O., Wellinger A. (2006). Biogas upgrading to vehicle fuel standards and grid injection. IEA Bioenergy Task.

[65] Juliani T. (2020). Is Biogas a “Green” Energy Source?. https://www.worldwildlife.org/blogs/sustainability-works/posts/is-biogas-a-green-energy-source.

[66] Nikolaidou C., Mola M., Papakostas S., Aschonitis V.G., Monokrousos N., Kougias P.G. (2024). The effect of anaerobic digestate as an organic soil fertilizer on the diversity and structure of the indigenous soil microbial and nematode communities. Environ. Sci. Pollut. Res. Int..

[67] Moestedt J., Westerholm M., Isaksson S., Schnürer A. (2019). Inoculum Source Determines Acetate and Lactate Production during Anaerobic Digestion of Sewage Sludge and Food Waste. Bioengineering.

[68] Siciliano A., Limonti C., Curcio G.M., Calabrò V. (2019). Biogas Generation through Anaerobic Digestion of Compost Leachate in Semi-Continuous Completely Stirred Tank Reactors. Processes.

[69] Rajput A.A., Sheikh Z. (2019). Effect of inoculum type and organic loading on biogas production of sunflower meal and wheat straw. Sustain. Environ. Res..

[70] Kulichkova G.I., Ivanova T.S., Köttner M., Volodko O.I., Spivak S.I., Tsygankov S.P., Blume Y.B. (2020). Plant Feedstocks and their Biogas Production Potentials. Open Agric. J..

[71] Aui A., Wang Y. (2025). Policy Support and Technology Development Trajectory for Renewable Natural Gas in the US. Renew. Energy.

[72] Gemechu F.K. (2020). Evaluating the potential of domestic animal manure for biogas production in Ethiopia. J. Energy.

[73] Lim J.W., Park T., Tong Y.W., Yu Z. (2020). The microbiome driving anaerobic digestion and microbial analysis. Advances in Bioenergy.

[74] USEPA Biosolids Technology Fact Sheet Multi-Stage Anaerobic Digestion DESCRIPTION. https://www.epa.gov/sites/default/files/2018-11/documents/multistage-anaerobic-digestion-factsheet.pdf.

[75] Issahaku M., Derkyi N.S.A., Kemausuor F. (2024). A systematic review of the design considerations for the operation and maintenance of small-scale biogas digesters. Heliyon.

[76] Abubakar A.M. (2022). Biodigester and feedstock type: Characteristic, selection, and global biogas production. J. Eng. Res. Sci..

[77] Sahu S.N., Gbagbo J.K.N., Aneke F.U. (1997). Comparative Evaluation of Different Types of Biogas Suitable for Tropical Country; Denmark. https://www.osti.gov/etdeweb/servlets/purl/618169.

[78] Jamal H., Loganathan M.K., Ramesh P.G., Singh M., Kumar G. (2023). Application of Multi-Criteria Decision-Making Tool for Choosing Right Biogas Plants: Process Controllability, Suitability, and Cost Perspectives in the Indian Context. International Conference on Recent Advances in Materials, Manufacturing and Thermal Engineering.

[79] Balloon Digester for Biogas Plants—Energypedia. energypedia.info. https://energypedia.info/wiki/Balloon_Digester_for_Biogas_Plants.

[80] del Agua I., Usack J.G., Angenent L.T. (2015). Comparison of semi-batch vs. continuously fed anaerobic bioreactors for the treatment of a high-strength, solids-rich pumpkin-processing wastewater. Environ. Technol..

[81] Liu S., Cao L., Xu F., Yang L., Li Y., Inalegwu O.S. (2021). Integration of algae cultivation to anaerobic digestion for biofuel and bioenergy production. Adv. Bioenergy.

[82] Sánchez M., Ruiz I., Soto M. (2023). Sustainable wastewater treatment using a new combined hybrid digester–constructed wetland system. J. Environ. Chem. Eng..

[83] ACIAR (2022). Exploring Potential Benefits of Biodigester Technologies. ACIAR. https://www.aciar.gov.au/media-search/blogs/exploring-potential-benefits-biodigester-technologies.

[84] Wilson L.P., Sharvelle S.E., Susan K. (2016). Enhanced AD performance via combined solids-and leachate-based hydrolysis reactor inoculation. Bioresour. Technol..

[85] Union of Concerned Scientists 2022 Annual Report. https://www.ucsusa.org/resources/2022-annual-report.

[86] (2023). Food Waste Digester Machine—Power Knot. Power Knot. https://powerknot.com/sea.

[87] C40 Knowledge Community. https://www.c40knowledgehub.org/s/article/Cities100-2019?language=en_US.

[88] de Souza S.N., Horttanainen M., Antonelli J., Klaus O., Lindino C.A., Nogueira C.E. (2014). Technical potential of electricity production from municipal solid waste disposed in the biggest cities in Brazil: Landfill gas, biogas and thermal treatment. Waste Manag. Res..

[89] Hojnacki A., Li L., Kim N., Markgraf C., Pierson D. (2011). Biodigester Global Case Studies. https://web.mit.edu/colab/pdf/papers/D_Lab_Waste_Biodigester_Case_Studies_Report.pdf.

[90] Davick A. (2023). Curbside Composting Coming to Five Boroughs Next Year, Mayor Says. Ny1.com. Spectrum News NY1. https://ny1.com/nyc/all-boroughs/politics/2023/01/29/curbside-composting-coming-to-five-boroughs-next-year--mayor-says.

[91] Karidis A. (2022). New Yorks Newtown Creek Wastewater Treatment Plant Anaerobic Co-Digestion. Waste360.com. https://www.waste360.com/wastewater/new-york-s-newtown-creek-wastewater-treatment-plant-revs-up-anaerobic-co-digestion-project.

[92] Brendlen K. (2024). National Grid’s “First of Its Kind” Fuel Project at Newtown Creek Treatment Plant Draws Ire over Cost, Delays and Malfunctions. Brooklyn Paper. https://www.brooklynpaper.com/fuel-project-newtown-creek-national-grid-ire/.

[93] (2023). Project: Salisbury, VT—Goodrich Family Farm. Vanguard Renewables. https://www.vanguardrenewables.com/projects/goodrich-family-farm.

[94] Vanguard’s Violations. No Digester. www.nodigester.com/lack-of-ethics.

[95] California Diary Digestors. https://www.dairycares.com/dairy-digesters.

[96] Dairy Digesters: Dairy Digesters in California: Creating Clean Energy. Dairy Cares. www.dairycares.com/dairy-digesters.

[97] Maryland Bioenergy Center—Jessup (MD). BTS Biogas. https://bts-biogas.com/en/maryland-bioenergy-center-jessup-md/.

[98] Shwe E. (2021). More Food Waste in Md. Will Be Diverted as a Large Anaerobic Digestion Facility Is Underway—Maryland Matters. Maryland Matters. https://marylandmatters.org/2021/07/19/more-food-waste-in-md-will-be-diverted-as-a-large-anaerobic-digestion-facility-is-underway/.

[99] Vermont Department of Environmental Conservation Vermont biodigestor. https://dec.vermont.gov/air-quality/permits/source-categories/anaerobic-digesters.

[100] Clement C. (2013). Vermont Technical College Holds Groundbreaking Ceremony for Biomass Anaerobic Digester—Vermont Technical College. Vermont Technical College. https://www.vtc.edu/vermont-technical-college-holds-groundbreaking-ceremony-for-biomass-anaerobic-digester/.

[101] Carpenter M., Verhar E., Lockhart I. (2022). Maine’s Largest Dairy Farm Will Soon Have the State’s First Natural Gas Digester, Fueled by Manure. Maine Public. WMEH. https://www.mainepublic.org/environment-and-outdoors/2022-07-06/maines-largest-dairy-farm-will-soon-have-the-states-first-natural-gas-digester-fueled-by-manure.

[102] Narayanan V. (2023). Chennai Start-Up Chuggs Food Waste to Generate Biogas. BusinessLine. https://www.thehindubusinessline.com/news/chennai-start-up-chuggs-food-waste-to-generate-biogas/article66996409.ece.

[103] KENPRO (2022). Biogas Plant Troubleshooting: Problems, Causes and Remedies. KENPRO. https://www.kenpro.org/biogas-plant-troubleshooting-problems-causes-and-remedies/.

[104] Mansharamani Y. (2024). Investigation into the Landfilling and Recovery of Wood and Wooden Pallets at US Landfills in 2021.

[105] Leal Filho W., Ribeiro P.C.C., Setti A.F.F., Azam F.M.S., Abubakar I.R., Castillo-Apraiz J., Tamayo U., Özuyar P.G., Frizzo K., Borsari B. (2023). Toward food waste reduction at universities. Environ. Dev. Sustain..

[106] Smith M.T. (2011). The Financial and Economic Feasibility of Biodigester Use and Biogas Production for Rural Households. Doctoral Dissertation.

[107] Stolecka K., Rusin A. (2021). Potential hazards posed by biogas plants. Renew. Sustain. Energy Rev..

[108] Gittelson P., Diamond D., Henning L., Payan M., Utesch L., Utesch N. (2021). The False Promises of Biogas: Why Biogas Is an Environmental Justice Issue. Environ. Justice.

[109] Alengebawy A., Ran Y., Osma A., Jin K., Samer M., Ai P. (2024). Anaerobic digestion of agricultural waste for biogas production and sustainable bioenergy recovery: A review. Environ. Chem. Lett..

[110] (2024). Effectively Ensuring Safety in Biogas Facilities—AZURA [Internet]. Azuraassociates.com. https://azuraassociates.com/ensuring-safety-in-biogas-facilities/.

[111] Schmidt Futures (2023). Feedstocks of the Future for a Circular U.S. Bioeconomy: A SUMMARY FROM A STAKEHOLDER CONVENING [Internet]. Foundation for Food and Agriculture Research. https://foundationfar.org/wp-content/uploads/2023/06/Feedstocks-of-the-Future-Convening-Report_FINAL.pdf.

